# Fluticasone propionate/salmeterol 250/50 μg versus salmeterol 50 μg after chronic obstructive pulmonary disease exacerbation

**DOI:** 10.1186/s12931-014-0105-2

**Published:** 2014-09-24

**Authors:** Jill A Ohar, Glenn D Crater, Amanda Emmett, Thomas J Ferro, Andrea N Morris, Ibrahim Raphiou, Peruvemba S Sriram, Mark T Dransfield

**Affiliations:** Wake Forest University Medical Center, 1 Medical Center Boulevard, Winston-Salem, NC USA; GlaxoSmithKline, Research Triangle Park, NC USA; North Florida/South Georgia Veterans Health System, University of Florida, Gainesville, FL USA; Lung Health Center University of Alabama, Birmingham, AL USA

## Abstract

**Background:**

Inhaled long-acting beta_2_ agonists used alone and in combination with an inhaled corticosteroid reduce the risk of exacerbations in patients with stable COPD. However, the relative efficacy of these agents in preventing recurrent exacerbations in those recovering from an initial episode is not known. This study compared the rate of COPD exacerbations over the 26 weeks after an initial exacerbation in patients receiving the combination of fluticasone propionate and salmeterol (FP/SAL) or SAL alone.

**Methods:**

Patients (n = 639) aged ≥40 years were randomized to either twice-daily inhaled FP/SAL 250/50 μg or SAL 50 μg. Primary, and secondary, endpoints were rates of recurrent severe, and moderate/severe, exacerbations of COPD. Lung function, health outcomes and levels of biomarkers of systemic inflammation were also assessed.

**Results:**

There was no statistically significant treatment difference in rates of recurrent severe exacerbations (treatment ratio 0.92 [95% CI: 0.58, 1.45]) and moderate/severe exacerbations (0.82 [0.64, 1.06]) between FP/SAL and SAL in the intent-to-treat population. Pre-dose morning FEV_1_ change from baseline was greater (0.10 L [0.04, 0.16]) with FP/SAL than SAL. No treatment difference was seen for other endpoints including patient-reported health outcomes and biomarker levels for the full cohort.

**Conclusions:**

No significant treatment difference between FP/SAL and SAL was seen in COPD exacerbation recurrence for the complete cohort. Treatment benefit with FP/SAL over SAL (treatment ratio 0.68 [0.47, 0.97]) was seen in patients having FEV_1_ ≥ 30% and prior exposure to ICS. No unexpected safety issues were identified with either treatment. Patients with the most severe COPD may be more refractory to treatment.

**Trial registration:**

ClinicalTrials.gov (identifier NCT01110200). This study was funded by GlaxoSmithKline (study number ADC113874).

**Electronic supplementary material:**

The online version of this article (doi:10.1186/s12931-014-0105-2) contains supplementary material, which is available to authorized users.

## Introduction

Exacerbations are clinically important events in COPD [1], becoming more frequent and more severe as airflow limitation worsens [2]. A frequent exacerbator phenotype independent of baseline FEV_1_ has also been identified [3]. Increased frequency of exacerbations has also been associated with an accelerated decline in lung function [4,5], worse health status [6,7], increased mortality and morbidity, and high healthcare costs [8]. Furthermore, exacerbations have been shown to exhibit temporal clustering and patients are more likely to suffer an exacerbation in the period immediately following an index exacerbation [9]. There is also an increased risk of co-morbid events associated with systemic inflammation in the aftermath of an exacerbation [10-12]. Reducing the frequency and recurrence of exacerbations is therefore a therapeutic priority in COPD [13].

Inhaled corticosteroid (ICS) and long-acting beta_2_ agonists (LABA) combination therapy has been found to reduce recurrence of COPD exacerbations and subsequent rehospitalization and mortality [14], and to significantly reduce rates of moderate or severe exacerbations, relative to treatment with LABA alone [15]. However, in previous large-scale studies of ICS/LABA therapy, randomization took place up to 1 year after the index exacerbation event [16,17].

In this study, patients with COPD received double-blind treatment, commencing within 14 days following an initial exacerbation, with either an ICS/LABA combination of fluticasone propionate/salmeterol (FP/SAL) in a single inhaler, or a LABA alone, SAL monotherapy. The aim of the study was to compare treatment effects on the rate of COPD exacerbations requiring hospitalization, and requiring treatment with oral corticosteroids (OCS) or OCS and antibiotics. Additional endpoints included measures of lung function and health status, incorporating EXACT-PRO (EXAcerbations of Chronic Pulmonary disease Tool – Patient Reported Outcome), a new measure of exacerbation frequency, severity and duration [18]. Levels of three inflammatory biomarkers, including high-sensitivity C-reactive protein (*hs*-CRP), Clara Cell secretory protein 16 (CC-16), and surfactant protein D (SP-D), were measured to investigate a possible association between systemic inflammation, exacerbation frequency [19] and severity of disease [20].

## Methods

### Study population

Male and female patients with COPD [21] aged ≥40 years were eligible for enrollment if they had recent (≤14 days) history of exacerbation requiring: *a)* hospitalization for ≤10 days; *b)* emergency room observation of duration ≥24 hours during which OCS/OCS + antibiotics treatment was administered; or *c)* physician’s office or emergency room visit of <24 hours duration with OCS/OCS + antibiotics treatment plus 6-month history of exacerbation-related hospitalization. Full details of inclusion and exclusion criteria, permitted and prohibited medications are provided in Additional file 1. Each participating patient provided written informed consent prior to study entry. The study was conducted in accordance with the Declaration of Helsinki and Good Clinical Practice guidelines, and approved by the applicable ethics committee or institutional review board at each site (Additional file 2).

### Study design

**Figure 1:**
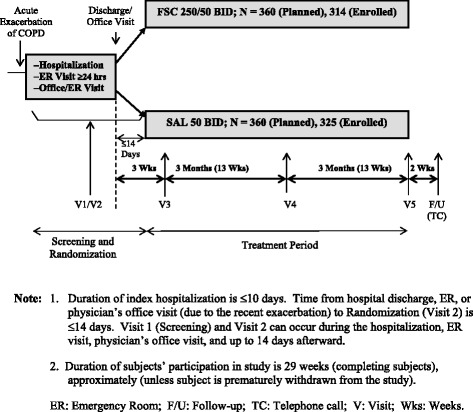
**Chronological schematic of experimental design.** Note: 1. Duration of index hospitalization is ≤10 days. Time from hospital discharge, ER, or physician’s office visit (due to the recent exacerbation) to Randomization (Visit 2) is ≤14 days. Visit 1 (Screening) and Visit 2 can occur during the hospitalization, ER visit, physician’s office visit, and up to 14 days afterward. 2. Duration of subjects’ participation in study is 29 weeks (completing subjects), approximately (unless subject is prematurely withdrawn from the study). ER: Emergency Room; F/U: Follow-up; TC: Telephone call; V: Visit; Wks: Weeks.

This was a randomized, double-blind, parallel-group, active-comparator study (GSK study ADC113874; ClinicalTrials.gov identifier NCT01110200) conducted in 81 centers in the United States, Argentina and Norway, from April 2010 to May 2012. Patients received FP/SAL 250/50 μg or SAL 50 μg for self-administration twice daily via DISKUS™ inhaler during a 21-day ‘stabilization period’ beginning within 14 days post-discharge and for a subsequent 26-week treatment period. Clinic visits were scheduled post-discharge: within 14 days; at 21 days; at 3 months; and at 6 months. Patients were randomized to study treatment within 14 days of discharge from hospital or emergency room, or of the physician’s office visit for the index exacerbation. A chronological diagram of the experimental design is presented in Figure 1. Patients requiring prolonged (protocol-defined as a period of up to 28 days) treatment with OCS and/or antibiotics during the stabilization period were to be withdrawn from the study.

Randomization (1:1) was according to a schedule, stratified by background tiotropium use and prior ICS use, generated by the sponsor using internally validated software (RandAll, GlaxoSmithKline, UK). Allocation of double-blinded study treatments was conducted using RAMOS (GlaxoSmithKline, UK), an interactive voice-response system.

### Efficacy analyses

The primary endpoint was the estimated annualized rate of exacerbations requiring hospitalization (severe exacerbations). The secondary endpoint was rate of exacerbations requiring treatment with OCS, antibiotics and/or hospitalization, alone and in combination (moderate or severe exacerbations). Exacerbations were identified by the worsening for at least two documented consecutive days of at least two of: dyspnea, sputum volume, sputum purulence, or at least one of these combined with sore throat, cold symptoms, fever, or increased cough or wheeze.

Other efficacy endpoints included time to first moderate or severe exacerbation; probability of all-cause premature withdrawal from the study; pre-dose morning FEV_1_; supplemental use of albuterol; changes in biomarker levels; and patient-reported health outcomes (CRQ-SAS; EXACT-PRO, Additional file 3).

### Post-Hoc subgroup analyses

*Post-hoc* analyses of exacerbation rate and spirometry data were performed for patient subgroups defined by baseline post-bronchodilator % predicted FEV_1_ (<30%/≥30%) and prior ICS use or concurrent tiotropium use. An additional subgroup analysis compared pre-dose FEV_1_ and questionnaire scores for patients experiencing ≥1 or 0 on-treatment exacerbations.

### Safety analyses

Adverse events (AEs) were documented by the study investigators at each on-treatment visit and on a follow-up call 2 weeks following completion of the study or discontinuation of study medication, and coded using MedDRA. Blood pressure and heart rate measurements were collected at each visit.

### Statistical analysis

All efficacy and safety analyses were performed in the intent-to-treat (ITT) population, consisting of all eligible patients randomized to study treatment. The study aimed to recruit an ITT population of 720 patients, which would provide 90% power to detect a treatment effect on the primary efficacy endpoint of 44% at the 0.05 significance level; this estimate was based on previously observed severe exacerbation rates (0.28–0.50) in patients with 1-year history of COPD-related hospitalization [16,17].

The primary and secondary efficacy endpoints were analyzed using a negative binomial regression model with terms for treatment group, pooled investigator, randomization stratum, and baseline % predicted FEV_1_. Log (time of treatment) was an offset variable. To account for multiple comparisons for several efficacy endpoints, a step-down statistical hierarchy was implemented. Statistical methods used to analyze other efficacy endpoints are detailed in Additional file 3.

## Results

### Patient disposition and baseline characteristics

**Figure 2:**
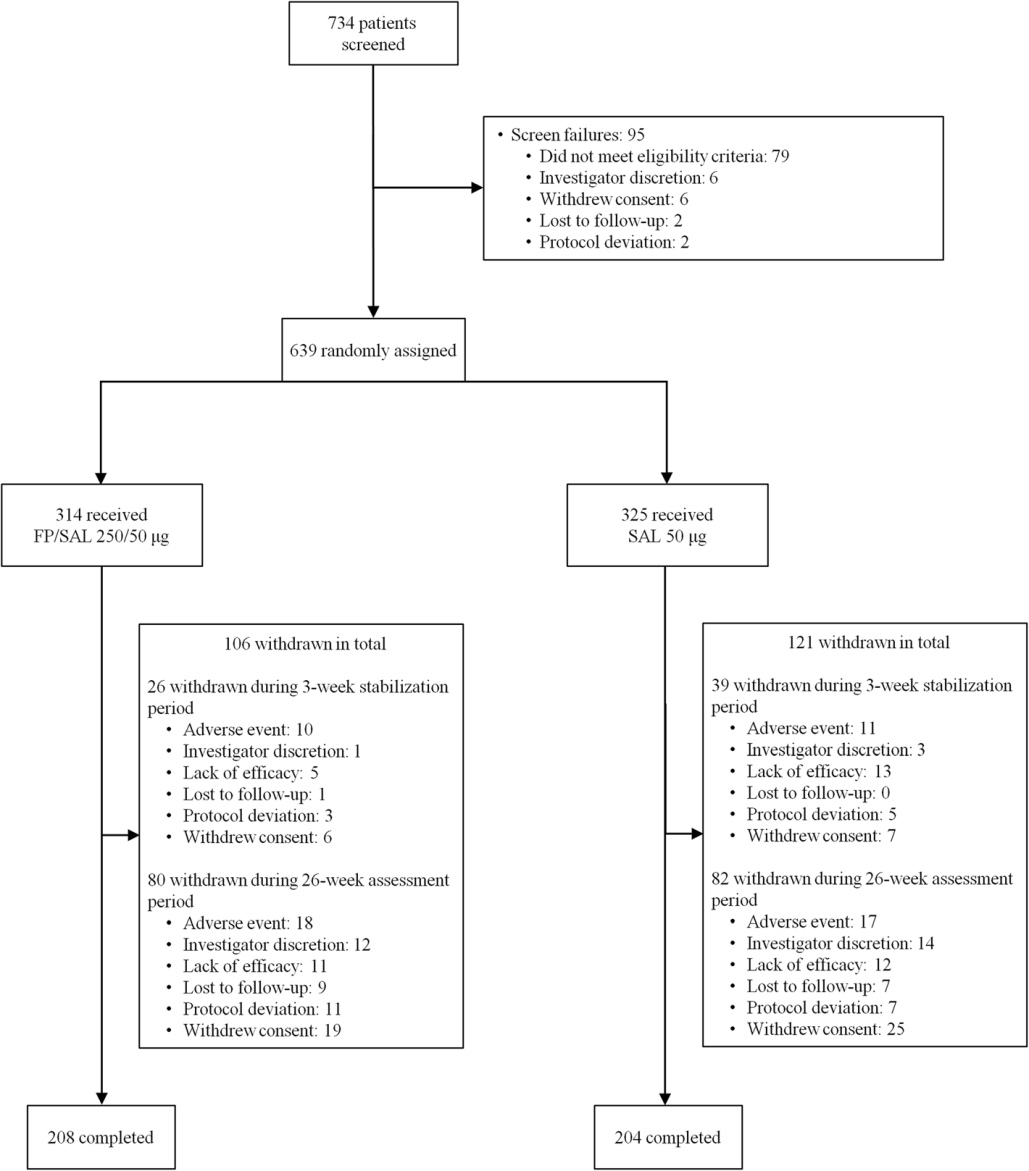
**Patient disposition and reasons for study withdrawal.** FP = fluticasone propionate; SAL = salmeterol.

Of 734 patients screened, 639 formed the ITT population (Figure 2). Patient demographics and baseline characteristics were well balanced between groups (Table 1).

**Table 1:** **Patient demographics and baseline characteristics (ITT Population)**

	**FP/SAL 250/50 μg (N = 314)**	**SAL 50 μg (N = 325)**	**Total (N = 639)**
Age, years	63.1 (9.15)	62.7 (9.30)	62.9 (9.22)
Female sex, n (%)	140 (45)	151 (46)	291 (46)
White race, n (%)	284 (90)	300 (92)	584 (91)
Duration of COPD, years	7.0 (5.7)	6.6 (5.2)	6.8 (5.4)
COPD type			
Chronic bronchitis (%)	114 (36)	129 (40)	243 (38)
Emphysema (%)	121 (39)	119 (37)	240 (38)
Both (%)	79 (25)	77 (24)	156 (24)
Smoking pack-years	52.0 (30.0)	56.3 (33.4)	54.2 (31.8)
Body mass index, kg/m^2^	28.0 (6.85)	28.3 (6.95)	28.2 (6.90)
Baseline pre-bronchodilator FEV_1_, L	1.08 (0.476)	1.14 (0.467)	1.11 (0.472)
Baseline % predicted FEV_1_	38.5 (14.82)	41.2 (16.85)	39.9 (15.93)
FEV_1_ % reversibility	15.1 (23.79)	12.1 (16.69)	13.6 (20.52)
Reversibility			
Non-reversible (%)	232 (74)	245 (76)	477 (75)
Reversible (%)	80 (26)	79 (24)	159 (25)

Mean (SD) unless otherwise stated. Reversibility testing was performed following subject self-administration of four puffs (360 μg) albuterol. COPD = chronic obstructive pulmonary disease; FEV_1_ = forced expiratory volume in 1 second; FP = fluticasone propionate; ITT = intent-to-treat; SAL = salmeterol; SD = standard deviation.

### Exacerbation results

No statistically significant treatment differences between FP/SAL and SAL in rates of recurrent severe or moderate/severe exacerbations were observed in the ITT population (severe exacerbations: FP/SAL 0.44, SAL 0.48, *P =* .710; moderate/severe exacerbations: FP/SAL 1.49, SAL 1.81, *P =* .136) (Table 2). Because of the step-down statistical hierarchy, all other analyses were interpreted descriptively.

**Table 2:** **Severe and moderate/severe exacerbations over 26 weeks of treatment following the 3-week stabilization period in the ITT population and patient subgroups**

	**FP/SAL 250/50 μg (N = 314)**	**SAL 50 μg (N = 325)**	**Ratio FP/SAL:SAL (95% CI)**	***P*** **Value**
Severe exacerbations, ITT population; n (%)				
Patients having exacerbation	43 (14)	39 (12)		
Number of exacerbations	50	51		
Mean annualized exacerbation rate	0.44	0.48	0.92 (0.58, 1.45)	.710
Moderate/severe exacerbations, ITT population; n (%)				
Patients having exacerbation	102 (32)	115 (35)		
Number of exacerbations	156	182		
Mean annualized exacerbation rate	1.49	1.81	0.82 (0.64, 1.06)	.136
Moderate/severe exacerbations, patient subgroups; n (%)				
Baseline post-bronchodilator % predicted FEV_1_ ≥ 30% and prior ICS use				
n	180	193		
Patients having exacerbation	49 (27)	66 (34)		
Number of exacerbations	74	106		
Mean annualized exacerbation rate	1.54	2.28	0.68 (0.47, 0.97)	NA
Baseline post-bronchodilator % predicted FEV_1_ ≥ 30% and no prior ICS use				
n	60	66		
Patients having exacerbation	21 (35)	24 (36)		
Number of exacerbations	38	34		
Mean annualized exacerbation rate	1.07	0.91	1.18 (0.69, 2.00)	NA
Baseline post-bronchodilator % predicted FEV_1_ ≥ 30% and concurrent tiotropium use				
n	88	95		
Patients having exacerbation	28 (32)	32 (34)		
Exacerbations	40	56		
Mean annualized exacerbation rate	1.00	1.48	0.67 (0.41, 1.11)	NA
Baseline post-bronchodilator % predicted FEV_1_ ≥ 30% and no concurrent tiotropium use				
n	152	164		
Patients having exacerbation	42 (28)	58 (35)		
Number of exacerbations	72	84		
Mean annualized exacerbation rate	1.88	2.22	0.85 (0.58, 1.24)	NA
Baseline post-bronchodilator % predicted FEV_1_ < 30%				
n	72	65		
Patients using concurrent tiotropium	33 (46)	30 (46)		
Patients having exacerbation	31 (43)	25 (38)		
Number of exacerbations	43	42		
Mean annualized exacerbation rate	2.78	2.84	0.98 (0.61, 1.57)	NA

Annualized rate estimates, ratio, CI and *P*-value are from a negative binomial regression model with terms for treatment, country, randomization stratum, baseline severity and time on treatment. CI = confidence interval; FEV_1_ = forced expiratory volume in 1 second; FP = fluticasone propionate; ICS = inhaled corticosteroid; ITT = intent-to-treat; NA = not applicable; SAL = salmeterol.

A *post-hoc* analysis of annualized exacerbation rates indicated that patients in a subgroup (n = 373) with baseline post-bronchodilator % predicted FEV_1_ ≥ 30% and history of prior ICS experienced fewer exacerbations with FP/SAL (mean annualized exacerbation rate: 1.54) than SAL (2.28) (treatment ratio 0.68 [0.47, 0.97]) (Table 2). A greater proportion of patients in subgroups having % predicted FEV_1_ < 30% relative to ≥30% used tiotropium during the study (46% vs. 37%).

**Figure 3:**
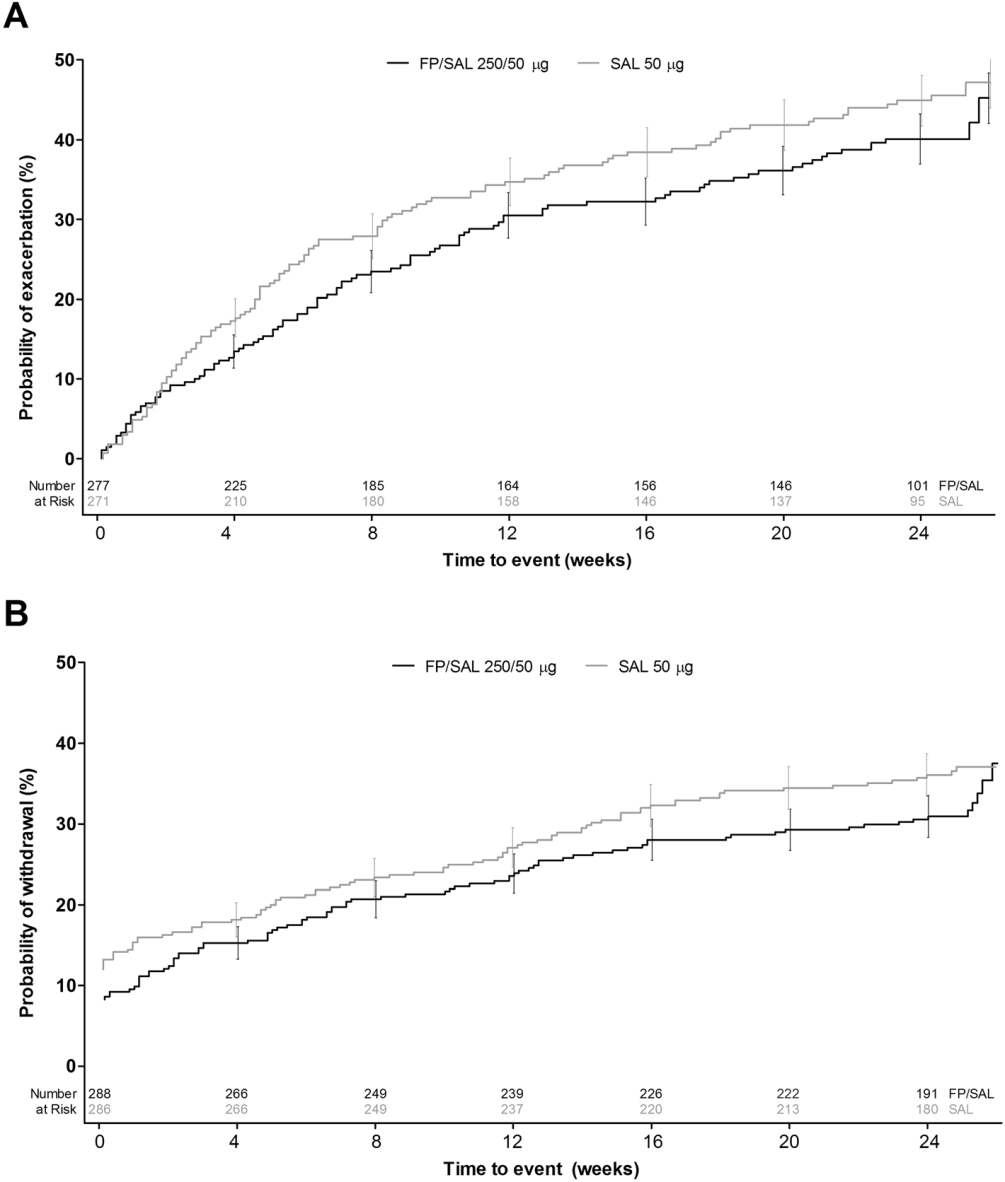
**Kaplan-Meier estimates for A) time to first COPD exacerbation requiring oral corticosteroids, antibiotics and/or hospitalization, and B) time to withdrawal from study, over 26 weeks of treatment following the 3-week stabilization period, ITT population.** COPD = chronic obstructive pulmonary disease; FP = fluticasone propionate; ITT = intent-to-treat; SAL = salmeterol.

**Figure 4:**
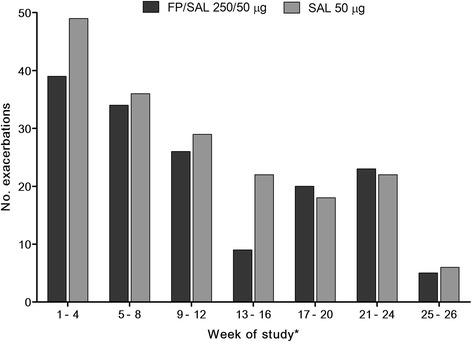
**Overall numbers of exacerbations by 4-week period, over 26 weeks of treatment following the 3-week stabilization period, ITT population.** *Weeks from end of 21-day stabilization period. FP = fluticasone propionate; ITT = intent-to-treat; SAL: salmeterol.

There was no overall indication of treatment differentiation for either time to first moderate/severe exacerbation, or withdrawal from the study during the treatment period (Table 3; Figure 3). Exacerbation frequency decreased as the treatment period progressed (Figure 4). In the first 4 weeks following the 3-week stabilization period, slightly more moderate/severe exacerbations occurred in the SAL arm than FP/SAL (49 vs. 39 exacerbations).

**Table 3:** **Kaplan-meier analysis of time to first moderate/severe exacerbation and to premature withdrawal of patients from the study (ITT Population)**

	**FP/SAL 250/50 μg**	**SAL 50 μg**	**Hazard Ratio (FP/SAL:SAL)**
	**(N = 314)**	**(N = 325)**	**(95% CI)**
Cumulative no. (%) patients with moderate/severe exacerbation	102 (32)	115 (35)	
% probability (95% CI) of moderate/severe exacerbation	45.2 (36.8, 54.5)	47.1 (40.6, 54.2)	0.83 (0.63, 1.09)
Cumulative no. (%) patients withdrawing from the study	98 (31)	119 (37)	
% probability (95% CI) of withdrawal from the study	31.7 (26.7, 37.3)	37.1 (32.0, 42.7)	0.87 (0.67, 1.13)

Hazard ratio and CI are from a Cox proportional hazards model with terms for treatment, country, randomization stratum and baseline severity. CI = confidence interval; FP = fluticasone propionate; ITT = intent-to-treat; SAL = salmeterol.

A post-hoc analysis of patient withdrawal during the 3-week stabilization period found that 65 (10%) patients withdrew from the study for any reason (FP/SAL 26 [8%], SAL 39 [12%]) (Table 4). Of these, 39 (6%) withdrew due to lack of efficacy or AE (FP/SAL 15 [5%], SAL 24 [7%]).

**Table 4:** **Study withdrawals during the 21-day stabilization period (ITT Population)**

**Patients withdrawing from study during 21-day stabilization period**	**FP/SAL 250/50 μg (N = 314)**	**SAL 50 μg (N = 325)**	**Nominal** ***P*** Value*
Any reason	26 (8%)	39 (15%)	.105
For lack of efficacy or adverse event	15 (5%)	24 (7%)	.158
For lack of efficacy	5 (2%)	13 (4%)	.062

All data are n (%).

*Nominal *P*-values are from Cochran-Mantel-Haenzel tests controlling for randomization stratum.

FP = fluticasone propionate; ITT = intent-to-treat; SAL = salmeterol.

### Other efficacy outcomes

**Figure 5:**
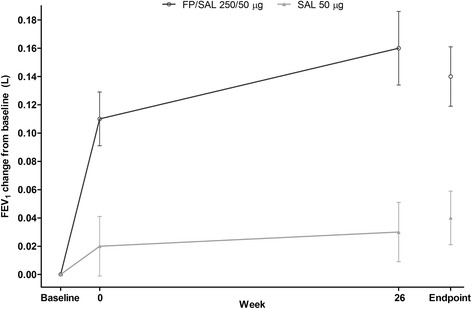
**Summary of pre-dose morning FEV**_**1**_ **during the 3-week stabilization period, over Weeks 0–26, and at endpoint, ITT population.** FEV_1_ = forced expiratory volume in one second; FP = fluticasone propionate; ITT = intent-to-treat; SAL = salmeterol.

Pre-dose morning FEV_1_ findings suggested a treatment difference in favor of FP/SAL, overall (Figure 5) and across patient subgroups (Table 5). A greater treatment effect of adding FP to SAL on FEV_1_ was seen in patients with post-bronchodilator % predicted FEV_1_ ≥ 30% not receiving concurrent tiotropium. There was no notable treatment difference in patients receiving concurrent tiotropium.

**Table 5:** **Pre-Dose FEV**_**1**_ **(L) Data, ITT population and patient subgroups**

			**FP/SAL 250/50 μg (N = 314)**	**SAL 50 μg (N = 325)**	**LS Mean Diff. (SE)**	**95% CI**
ITT population						
Baseline	FEV_1_	n	313	325		
		Mean (SE)	1.08 (0.027)	1.14 (0.026)		
Endpoint	FEV_1_	n	281	271		
		Mean (SE)	1.22 (0.034)	1.18 (0.031)		
	Change from baseline	n	280	271	0.10 (0.028)	(0.04, 0.16)
		Mean (SE)	0.14 (0.021)	0.04 (0.019)		
Baseline post-bronchodilator % predicted FEV_1_ ≥ 30% and no prior ICS use						
Baseline	FEV_1_	n	60	66		
		Mean (SE)	1.28 (0.057)	1.26 (0.054)		
Endpoint	FEV_1_	n	57	59		
		Mean (SE)	1.52 (0.083)	1.39 (0.072)		
	Change from baseline	n	57	59	0.13 (0.083)	(−0.04, 0.29)
		Mean (SE)	0.25 (0.064)	0.13 (0.051)		
Baseline post-bronchodilator % predicted FEV_1_ ≥ 30% and prior ICS use						
Baseline	FEV_1_	n	180	193		
		Mean (SE)	1.20 (0.033)	1.26 (0.031)		
Endpoint	FEV_1_	n	162	159		
		Mean (SE)	1.30 (0.039)	1.28 (0.036)		
	Change from baseline	n	162	159	0.10 (0.036)	(0.03, 0.17)
		Mean (SE)	0.12 (0.027)	0.01 (0.025)		
Baseline post-bronchodilator % predicted FEV_1_ ≥ 30% and concurrent tiotropium use						
Baseline	FEV_1_	n	88	95		
		Mean (SE)	1.17 (0.044)	1.19 (0.041)		
Endpoint	FEV_1_	n	79	71		
		Mean (SE)	1.24 (0.055)	1.21 (0.052)		
	Change from baseline	n	79	71	0.02 (0.053)	(−0.09, 0.12)
		Mean (SE)	0.07 (0.037)	0.06 (0.041)		
Baseline post-bronchodilator % predicted FEV_1_ ≥ 30% and no concurrent tiotropium use						
Baseline	FEV_1_	n	152	164		
		Mean (SE)	1.25 (0.037)	1.31 (0.034)		
Endpoint	FEV_1_	n	140	147		
		Mean (SE)	1.43 (0.047)	1.36 (0.042)		
	Change from baseline	n	140	147	0.16 (0.044)	(0.07, 0.25)
		Mean (SE)	0.21 (0.035)	0.03 (0.027)		
Baseline post-bronchodilator % predicted FEV_1_ < 30%						
Baseline	FEV_1_	n	72	65		
		Mean (SE)	0.64 (0.032)	0.63 (0.022)		
Endpoint	FEV_1_	n	60	53		
		Mean (SE)	0.72 (0.046)	0.65 (0.025)		
	Change from baseline	n	60	53	0.06 (0.037)	(−0.01, 0.14)
		Mean (SE)	0.08 (0.023)	0.01 (0.028)		

LS mean difference, SE and CI are from an ANCOVA model with terms for treatment, country, randomization stratum and baseline value. LS mean diffs. are calculated as FP/SAL 250/50 μg–SAL 50 μg. ANCOVA = analysis of covariance; CI = confidence interval; FEV_1_ = forced expiratory volume in 1 second; FP = fluticasone propionate; ICS = inhaled corticosteroid; ITT = intent-to-treat; LS = least squares; SAL = salmeterol; SE = standard error.

There was no treatment difference in rescue medication use (data not shown) or for any health outcome comparisons (Additional file 4: Table S1) at study endpoint. In a secondary analysis, patients who did not experience on-treatment exacerbations showed significantly more improvement in the dyspnea domain of CRQ-SAS [22] and in EXACT-PRO total score at study endpoint than those who did. There were also some indications of greater improvement in other CRQ-SAS domains (Additional file 5: Table S2). However, no difference in change from baseline pre-dose FEV_1_ was observed between patients who did not experience on-treatment exacerbations and those who did (data not shown).

Levels of all three inflammatory biomarkers were elevated at baseline and remained elevated throughout the 26-week assessment period; no treatment effect on biomarker levels was observed (Additional file 6: Table S3). No meaningful associations between biomarker levels and occurrence of on-treatment exacerbation were observed. Treatment of the index events may have altered the initial level of the biomarker assay.

### Safety outcomes

AE and serious AE frequencies were comparable between the treatment groups (Table 6). The incidence of pneumonia (FP/SAL: 4%, SAL: 3%) was consistent with previous observations from FP/SAL exacerbation studies [23]. Seven fatal AEs occurred during the treatment period (FP/SAL: 4; SAL: 3) (Additional file 7).

**Table 6:** **Number (%) of On-treatment and post-treatment AEs and SAEs**

	**FP/SAL 250/50 μg (N = 314)**	**SAL 50 μg (N = 325)**
AEs (on-treatment)	185 (59)	205 (63)
COPD	47 (15)	51 (16)
Headache	19 (6)	19 (6)
Upper respiratory tract infection	15 (5)	20 (6)
Back pain	10 (3)	13 (4)
Diarrhea	11 (4)	10 (3)
Edema peripheral	6 (2)	14 (4)
Nausea	5 (2)	13 (4)
Treatment-related AEs (on-treatment)	19 (6)	22 (7)
AEs leading to withdrawal from study	29 (9)	33 (10)
SAEs (on-treatment)	75 (24)	82 (25)
SAEs (post-treatment)	16 (5)	8 (2)
Fatal SAEs (on-treatment)	4 (1)	3 (<1)
Pneumonia AEs (all)	13 (4)	10 (3)

Adverse events occurring in ≥2% of patients in either treatment group shown. AE = adverse event; COPD = chronic obstructive pulmonary disease; FP = fluticasone propionate; SAE = serious adverse event; SAL = salmeterol.

## Discussion

No statistically significant treatment difference in the primary endpoint of this study, the rate of COPD exacerbations requiring hospitalization, assessed over six months, was achieved. The lack of exacerbation reduction was noted despite the positive spirometric data supporting the clinical benefit of the FP/SAL compared with SAL. FP/SAL has previously been shown to reduce the frequency of moderate/severe exacerbations compared with SAL in patients with a prior history of exacerbations in parallel 52-week studies [16,17].

The objective of the study was to evaluate the treatment effects of FSC 250/50 mcg BID in comparison to salmeterol 50 mcg BID, both via DISKUS, on exacerbations of COPD requiring treatment with oral corticosteroids, antibiotics, and/or hospitalization (alone and in combination), over a 29-week treatment period. The primary efficacy measure was the rate of exacerbation requiring hospitalization. Although treatment intervention with ICS/LABA combination therapy was known to reduce the rate of exacerbations more effectively that LABA alone therapy in clinically stable patients with a history of exacerbation, we aimed to investigate the potential benefit of an early treatment intervention immediately following a moderate to severe exacerbation of COPD. The potential benefits of this treatment paradigm had not been studied previously, is not widely accepted but has major clinical relevance given the increasing focus on hospital readmission, particular in the United States. While many patients who experience an acute exacerbation of COPD recover quickly, mortality exceeds 10% during hospitalization, increases to 25-40% during the year after hospital discharge [24] and 63% of discharged patients experience subsequent exacerbations and readmissions [25]. Other data show that although 75% of those patients who survive regain their basal pulmonary function within five weeks post-hospitalization, 7% of patients do not recover even after five months following the acute episode [20,26]. Hence, the study was initiated in an attempt to address these clinical outcomes.

In the 3-year TORCH study, in which 57% of subjects had an exacerbation within the preceding year, adding FP to SAL resulted in a significant reduction in moderate/severe exacerbations and in exacerbations requiring OCS, but not in severe exacerbations requiring hospitalization. Concurrent long-acting bronchodilators (including tiotropium) were not permitted in these earlier studies, but were allowed in the present trial and may have impacted the results discussed below. The findings of a meta-analysis of 18 randomized trials of ICS/LABA combination therapy [27] concurred with those of TORCH, identifying a significant benefit of the combination on moderate, but not severe, exacerbations.

Unlike the studies described above, our study was designed to investigate the effect on severe exacerbation rates of ICS intervention in the period shortly after an acute COPD exacerbation. This endpoint is of particular interest to United States clinicians, as 30-day re-admission following exacerbation will be subject to financial penalties imposed by the Centre for Medicare and Medicaid Services under the Hospital Readmissions Reduction Program [28]. All patients in this study had exacerbation requiring hospitalization and/or treatment with OCS within the month prior to randomization.

Our findings are consistent with previous observations of a high-risk period for recurrence within 8 weeks of index exacerbation [9]. To allow sufficient time for patients to recover from the index exacerbation before the start of outcome measure assessment, patients experiencing an exacerbation during the 21-day stabilization period were to be withdrawn and those exacerbations were not included in the efficacy analyses. A potential confounding factor was that more patients receiving SAL than FP/SAL withdrew from the study during the 21-day stabilization period for any reason including lack of efficacy and/or AE. More patients receiving SAL, compared with FP/SAL, experienced a moderate/severe exacerbation in the first month of treatment. These observations may indicate a potential benefit of immediate post-event treatment with ICS/LABA maintenance therapy in reducing the likelihood of hospital readmission in the 30 days post-event. However, the study was not designed to test this hypothesis; furthermore, a substantial proportion (>60%) of readmissions of patients initially hospitalized for COPD are due to factors other than COPD recurrence [29] and hence may not be influenced by choice of COPD maintenance therapy. The safety profiles of the two treatments are consistent with previous findings [30].

A *post-hoc* analysis of moderate/severe exacerbation rates identified that patients with greater lung function (% predicted FEV_1_ ≥ 30%) and prior use of ICS receiving FP/SAL versus SAL had 32.3% lower annualized exacerbation rate. This effect size is similar to that observed previously in 52-week studies of FP/SAL and SAL in which concurrent tiotropium was not permitted [16,17]. These findings suggest a possibility of achieving a significant reduction in recurrence by targeting post-exacerbation treatment at subgroups of patients who display defined characteristics associated with recurrence or ICS responsiveness [31,32]. They also suggest a greater potential effect on risk of recurrence of exacerbations following withdrawal of ICS therapy, an observation consistent with previously reported findings [33,34].

Clinically meaningful improvement from baseline in pre-dose FEV_1_ was seen with FP/SAL (+140 mL) but not SAL (+40 mL). No treatment difference was observed in the third of patients using concurrent tiotropium. The inclusion of patients using concurrent tiotropium in the cohort may have confounded the treatment effect. A one-year study of patients with a history of prior exacerbation within the preceding year found that adding SAL to tiotropium, with and without FP, did not significantly reduce exacerbation rate overall, although a significant reduction in severe exacerbation rate was observed with SAL + FP + tiotropium triple therapy compared to treatment with tiotropium alone [35]; however, this study was under powered to demonstrate an effect on this variable; whereas adding tiotropium to ICS/LABA combination therapy conferred significant benefits in mortality, hospitalizations, and OCS use in a retrospective cohort analysis [36]. Furthermore, a two-year study comparing FP/SAL with tiotropium on exacerbation rate did not find a significant treatment difference [37].

No treatment difference in health outcomes (CRQ-SAS or EXACT-PRO) was seen. Levels of inflammatory biomarkers, heightened across the cohort as anticipated due to the index exacerbation event [38], did not decrease substantially over the treatment period, and no treatment difference was observed. Both systemic inflammation and airway inflammation are associated with COPD exacerbations [39]. Although the persistence of inflammatory biomarkers subsequent to exacerbation has been reported [40], no clear relationship between biomarker levels and on-treatment exacerbation was found. The persistence of high biomarker levels across the study cohort over the 6-month study was an unexpected finding requiring further investigation, but may be reflective of disease severity and systemic inflammation.

Cross-cohort variables and challenges in the recruitment of patients shortly after an exacerbation were evident in this study. Recruitment was complicated by significant co-morbidities found in the target cohort, which frequently were cause for exclusion, by the difficulty of coordinating patient hospitalization, discharge and consent for study participation, and by the limited availability of investigators with both outpatient and inpatient practices qualifying them to participate in the study. The identification of such recruitment issues emphasizes the need for careful cohort definition in future studies of the timely treatment of COPD exacerbation risk. Defining eligibility criteria on the basis of prior treatment with ICS may help to identify a steroid-responsive cohort. The observation of baseline FEV_1_ below a defined threshold may help identify patients who are less likely to respond to treatment. Another factor that may have affected responsiveness to treatment was the unexpectedly low exacerbation rate seen in both study arms, possible explanations for which include the use of concurrent tiotropium by patients and temporal improvements in patient care. While the results of this study were negative, the implementation of lessons herein learned may result in future studies being appropriately powered to detect a statistically significant treatment effect on rehospitalization rate, to assist clinicians to identify COPD phenotypes, including the presence or absence of common COPD co-morbidities, most likely to benefit from ICS/LABA intervention immediately following an exacerbation [41].

Although the primary and other pre-specified outcomes of this study did not show statistical significance, the data support previous findings of significant beneficial effect of combination therapy on lung function [15]. Data on withdrawals during the 21-day stabilization period and exacerbations during the first month of the 26-week treatment period suggest a potential benefit of ICS/LABA in the period immediately following an exacerbation, and may warrant further clinical investigation. It is worth noting the findings of a post-hoc analysis, which showed that the rate of on-treatment study withdrawal due to lack of efficacy in the SAL arm (4%; n = 13) was approximately double that observed in the FP/SAL arm (2%; n = 5); however, the difference was not statistically significant (p = 0.062). The outcome of *post-hoc* subgroup analysis, which identified a greater effect of ICS/LABA on exacerbation rates in patients with predicted FEV_1_ ≥ 30% and prior use of ICS, underscored the potential importance of considering patient-specific factors in post-exacerbation treatment decisions, and suggested an ICS withdrawal effect [33].

The findings of this study highlight the complexity of studying interventions in the post-exacerbation period and emphasize the impact that patient-specific clinical factors and concomitant medication use may have on outcomes. In addition, future studies should be designed to capture recurrent or continued exacerbations in the immediate recovery period.

## Additional files

Additional file 1:
**Study inclusion and exclusion criteria, and permitted and prohibited medications.**
Additional file 2:
**Institutional Review Board details for study sites.**
Additional file 3:
**Statistical methods used in the analysis of other efficacy endpoints.**
Additional file 4: Table S1. CRQ-SAS Change from Baseline at 3 + 26-Week Study Endpoint and EXACT-PRO Total Score and Components at Study Endpoint, ITT Population. Additional file 5: Table S2. Change from Baseline CRQ-SAS Domain Scores and EXACT-PRO Total Scores at Treatment Period Weeks 13 and 26 and at 26-Week Endpoint for Patients Having 0 or ≥1 On-Treatment Exacerbations, ITT Population. Additional file 6: Table S3. Change from Baseline at 3 + 26-Week Study Endpoint for Biomarkers of Systemic Inflammation, ITT Population. Additional file 7:
**On- and post-treatment serious and fatal adverse events and incidences of pneumonia.**

## Abbreviations

AE: Adverse event
AECOPD: Acute exacerbation of COPD
ANCOVA: Analysis of covariance
ATS: American thoracic society
CC-16: Clara cell secretory protein 16
CHF: Congestive heart failure
CI: Confidence interval
COPD: Chronic obstructive pulmonary disease
CRQ-SAS: Chronic respiratory disease questionnaire - self-administered standardized format
CT: Computed tomography
ECG: Electrocardiogram
ER: Emergency room
EXACT-PRO: Exacerbations of chronic pulmonary disease tool - patient reported outcome
FEV_1_: Forced expiratory volume in 1 second
FP: Fluticasone propionate
FP/SAL: Fluticasone propionate/salmeterol combination
FVC: Forced vital capacity
*hs*-CRP: High-sensitivity C-reactive protein
ICS: Inhaled corticosteroids
ITT: Intent-to-treat
LABA: Long-acting beta_2_ agonist
LS: Least squares
MedDRA: Medical dictionary for regulatory activities
NA: Not applicable
OCS: Oral corticosteroids
SAE: Serious adverse event
SAL: Salmeterol
SD: Standard deviation
SE: Standard error
SP-D: Surfactant protein D
