# Supplementary material for: Fluticasone propionate/salmeterol 250/50 μg versus salmeterol 50 μg after chronic obstructive pulmonary disease exacerbation
Source: Respir Res. 2014 Sep 24;15(1):105. doi: 10.1186/s12931-014-0105-2 (PMC4176847; doi:10.1186/s12931-014-0105-2)
Supplement: Additional file 1: — Study inclusion and exclusion criteria, and permitted and prohibited medications. [file 12931_2014_105_MOESM1_ESM.docx]

**Fluticasone Propionate/Salmeterol 250/50µg Versus Salmeterol 50µg After Chronic Obstructive Pulmonary Disease Exacerbation**

**Authors:** *Jill A. Ohar, MD; Glenn D. Crater, MD; Amanda Emmett, MS; Thomas J. Ferro, MD; Andrea N. Morris, BSN; Ibrahim Raphiou, PhD; P.S. Sriram, MD; and Mark T. Dransfield, MD*

Supplemental Material

# Additional file 1: Study inclusion and exclusion criteria, and permitted and prohibited medications

**Inclusion Criteria**

Patients eligible for enrollment in this study were required to meet all of the following criteria:

1. Male or female of ≥40 years of age at screening.
2. Current or former smokers with a ≥10 pack-year cigarette smoking history [number of pack years = (number of cigarettes per day / 20) X number of years smoked (e.g., 10 pack-years is equal to 20 cigarettes per day for 10 years, or 10 cigarettes per day for 20 years]. Former smokers are defined as those who have quit smoking for at least 3 months prior to the screening visit.
3. Classification as part of one of the following populations:

- Patients hospitalized for a duration not exceeding 10 days due to an acute exacerbation of chronic obstructive pulmonary disease (AECOPD), eligible patients must be randomized within 14 days post-discharge.
- Patients with COPD who were treated with oral corticosteroids (OCS) or OCS and antibiotics, and held for observation in the emergency department (i.e.. emergency room [ER]) for at least 24 h due to an AECOPD; eligible patients must be randomized within 14 days post-discharge.
- Patients who received OCS or OCS and antibiotics for treatment of an AECOPD during a physician’s office visit or ER visit of less than 24 h in the 14 days prior to randomization, and who have been hospitalized within the previous 6 months due to an AECOPD.

1. Clinical diagnosis of COPD (for at least 6 months). The following definition of COPD from the American Thoracic Society (ATS) will be used: COPD is a disease state characterized by the presence of airflow obstruction due to chronic bronchitis or emphysema; the airflow obstruction is generally progressive, may be accompanied by airway hyper-reactivity, and may be partially reversible.
2. Documented evidence (within a year prior to Visit 1) in the medical chart of spirometry confirming the diagnosis of COPD and/or spirometry performed prior to randomization (Visit 2) that confirms pre-bronchodilator forced expiratory volume in 1 second (FEV_1_)/forced vital capacity (FVC) ratio <0.70 and pre-bronchodilator FEV_1_ <70% of predicted.
3. Review and subject’s completion of written informed consent: a subject-signed and dated written informed consent (form) must be obtained prior to any study procedure, and the subject must be willing to comply with all the requirements of the study protocol.
4. Patients were able to read, comprehend, and record information in the country-specific language presented (e.g., English if presented in English, Spanish if presented in Spanish, Norwegian if presented in Norwegian, etc.).

Females of childbearing potential were required to commit to the consistent and correct use of an acceptable method of birth control starting on the day of Visit 1, throughout the clinical trial, and for a period after the trial to account for elimination of the drug (minimum of 6 days).

**Exclusion Criteria**

Patients meeting any of the following criteria were not enrolled in the study:

1. Diagnosis of pneumonia, congestive heart failure (CHF), or other complicating co-morbid condition while hospitalized within the last 6 months for an exacerbation of COPD.
2. Historical or current evidence of clinically significant uncontrolled disease including, but not limited to, those listed below. Significant is defined as any disease that, in the opinion of the investigator, would put the safety of the subjects at risk through study participation, or which would affect the safety analysis or other analyses if the disease/condition exacerbated during the study.

- A previous lung resection surgery (e.g., lobectomy, pneumonectomy, etc) within the year preceding Visit 1 (Screening)
- Asthma as primary diagnosis
- Lung cancer
- Cystic fibrosis, pulmonary fibrosis, active tuberculosis, or sarcoidosis
- Clinically significant cardiac arrhythmias
- Uncontrolled hypertension
- Unstable angina
- Current malignancy or a previous history of cancer in remission for <5 years (localized basal cell or squamous cell carcinoma of the skin that has been resected is not excluded)
- Uncontrolled diabetes mellitus
- Uncontrolled hyperthyroidism or hypothyroidism
- Immunologic compromise
- Cushing’s or Addison’s disease

1. An abnormal 12-lead electrocardiogram (ECG) at Visit 1 (Screening) deemed to be clinically significant by the investigator.
2. A chest X-ray or computed tomography (CT) scan performed in the 6 months preceding Visit 1 that revealed evidence of clinically significant abnormalities not believed to be due to the presence of COPD. If the subject does not have a record of a chest X-ray, one must be obtained and reviewed prior to randomization.
3. Female patients with a positive urine pregnancy test at Visit 1.
4. Any infirmity, physical disability, or geographic location that would limit compliance for scheduled visits.
5. Any adverse reaction, immediate or delayed, hypersensitivity to any beta-agonist, sympathomimetic drug, or corticosteroid including any components of the study drug formulations.
6. Limited ability to provide a valid informed consent due to psychiatric disease, intellectual deficiency, poor motivation, current substance abuse (including illicit drugs and alcohol), or neurologic disorders that might interfere with completion of study procedures or hearing problems that may impede effective communication.
7. Study site staff (i.e., participating investigator, sub-investigator, study coordinator, employee of the participating investigator) or family members of site staffs.

The use of medications that were contraindicated for the study drugs was not permitted.

**Permitted medications and non-drug therapies**

All background COPD medications, with the exception of inhaled corticosteroids (ICS) and long-acting beta_2_ agonists (LABA), alone or in combination, were allowed, including the following:

- Albuterol was provided for use as relief medication for use on an as needed basis throughout the study but was to be withheld for at least 6 h prior to all study visits where pulmonary function testing was performed. If a subject had taken albuterol within 6 h prior to pulmonary function testing, the visit was rescheduled.
- Short courses of OCS were permitted for acute treatment of exacerbation of COPD, not to exceed 14 days.
- Tiotropium therapy started prior to, and during, the index exacerbation was allowed during the study. However, tiotropium therapy scheduled to start after randomization and during the treatment period of the study was not be permitted. Assignment to blinded study medication was to be stratified based on tiotropium use to ensure equal numbers of subjects using the drug in each treatment group.
- Antihistamines, nasal decongestants and/or other intranasal medications for the treatment of rhinitis were allowed.
- Beta-blockers were allowed provided study subjects were on a stable dose for at least 30 days prior to the screening visit (Visit 1) and this dose was maintained throughout the study.
- Beta-blocker eye drops were permitted.
- Immunotherapy for the treatment of allergies was allowed.
- Flu shots were allowed.
- Intranasal corticosteroids were allowed
- Intranasal cromolyns or nedocromil were allowed

Use of systemic corticosteroids for non-respiratory related medical treatment was permitted; however, the investigator were required to notify GlaxoSmithKline or designee if this occurred in order to determine subject eligibility.

Use of systemic antibiotics for non-respiratory related medical treatment was permitted.

Oxygen therapy was permitted during the study.

The following medications were permitted but the investigators were advised to use with extreme caution as they may potentiate the effects of fluticasone propionate (FP)/salmeterol (SAL) or SAL on the vascular system:

- monoamine oxidase inhibitors
- tricyclic antidepressants

Participation in a pulmonary rehabilitation program was allowed provided the pulmonary rehabilitation program was not initiated within 8 weeks of Visit 1. In addition, subjects who were enrolled in a pulmonary rehabilitation program at study start should have maintained participation in the program for the duration of the study.

Herbal therapies were allowed and their use recorded.

**Prohibited medications and non-drug therapies**

Use of concomitant inhaled corticosteroids and long-acting Beta2-agonists (other than study drug) was not permitted during the study treatment period. Prohibited medications and their exclusion periods are delineated in the table below.

| **Medication** | **Exclusion Period** |
| --- | --- |
| Inhaled corticosteroids  (e.g., fluticasone propionate) | Use not permitted during the study treatment period |
| Systemic corticosteroids (oral or intravenous) | Permitted at study entry, permitted for use of short courses for treatment of COPD exacerbations, not to exceed 14 days |
| Long-acting beta_2_-agonists  (e.g., salmeterol or formoterol) | Use not permitted during the study treatment period |
| ICS/LABA combination product  (e.g., Advair) | Use not permitted during the study treatment period |
| Tiotropium | Use not to be **started** during study treatment period (permitted if started prior to randomization) |
| Strong inhibitors of the cytochrome p450 Cyp 3A4 (e.g., ritonavir and ketoconazole) | Use not permitted during the study treatment period |
| Other investigational medication(s) | Use not permitted within 30 days prior to Visit 1 and Use not permitted during the study |
