# Supplementary material for: Fluticasone propionate/salmeterol 250/50 μg versus salmeterol 50 μg after chronic obstructive pulmonary disease exacerbation
Source: Respir Res. 2014 Sep 24;15(1):105. doi: 10.1186/s12931-014-0105-2 (PMC4176847; doi:10.1186/s12931-014-0105-2)
Supplement: Additional file 2: — Institutional Review Board details for study sites. [file 12931_2014_105_MOESM2_ESM.docx]

**Fluticasone Propionate/Salmeterol 250/50µg Versus Salmeterol 50µg After Chronic Obstructive Pulmonary Disease Exacerbation**

**Authors:** *Jill A. Ohar, MD; Glenn D. Crater, MD; Amanda Emmett, MS; Thomas J. Ferro, MD; Andrea N. Morris, BSN; Ibrahim Raphiou, PhD; P.S. Sriram, MD; and Mark T. Dransfield, MD*

Supplemental Material

# Additional file 2: Institutional Review Board details for study sites

**LIST OF INVESTIGATORS AND IECS/IRBS FOR ADC113874**

| **Investigator** | **Sub-Investigator** | **Investigator** | **Description of Research** | **Name of IEC/IRB Committee,** |  |
| --- | --- | --- | --- | --- | --- |
|  |  | **no./Center no.** | **Facility, Hospital/ Institution,** | **Address, Committee Chair** |  |
|  |  |  | **and Address** |  |  |
|  |  |  |  |  |  |
| **Argentina** |  |  |  |  |  |
|  |  |  |  |  |  |
| Abouzed, Roberto A. | Peirano, Hebe. MD | 238971/091554 | Asociación Española de | Buenos Aires, Larrea 1381, |  |
| MD | Sala, Alfredo. MD |  | Socorros Mutuos, Rivadavia 818, | Ciudad Autonoma de Buenos |  |
|  |  |  | Comodoro Rivadavia, Chubut, | Aires, C1117ABK, Argentina |  |
|  |  |  | U9000AKX, Argentina | Chairperson: Barclay, Carlos |  |
|  |  |  |  |  |  |
|  |  |  |  |  |  |
| Bustamante Labarta, | Elías, Enzo. MD | 207266/091406 | Instituto de Cardiologia de Jujuy, | Buenos Aires, Larrea 1381, |  |
| Gustavo F. MD | Surin García, Emilio. MD |  | Belgrano 340-4600, San | Ciudad Autonoma de Buenos |  |
|  |  |  | Salvador de Jujuy, Argentina | Aires, C1117ABK, Argentina |  |
|  |  |  |  | Chairperson: Barclay, Carlos |  |
|  |  |  |  |  |  |
| Cantero, Maria Cecilia. | Larivey, Virginia A. MD | 205796/090348 | Centro de Investigaciones | Buenos Aires, Larrea 1381, |  |
| MD |  |  | Clinicas del Litoral S.R.L, | Ciudad Autonoma de Buenos |  |
|  |  |  | Rivadavia 3363, Santa Fe, Santa | Aires, C1117ABK, Argentina |  |
|  |  |  | Fe, 3000, Argentina | Chairperson: Barclay, Carlos |  |
|  |  |  |  |  |  |
|  |  |  |  |  |  |

| **Investigator** | **Sub-Investigator** | **Investigator** | **Description of Research** | **Name of IEC/IRB Committee,** |
| --- | --- | --- | --- | --- |
|  |  | **no./Center no.** | **Facility, Hospital/ Institution,** | **Address, Committee Chair** |
|  |  |  | **and Address** |  |
|  |  |  |  |  |
| Charquero, Leonardo. | Díaz, Samanta. MD | 238368/091429 | Hospital Distrital Lago Argentino, | Buenos Aires, Larrea 1381, |
| MD | Sabio, Rodrigo. MD |  | Calle Pres Gral J. A. Roca 1487, | Ciudad Autonoma de Buenos |
|  |  |  | El Calafate, Santa Cruz, | Aires, C1117ABK, Argentina |
|  |  |  | Z9405CJM, Argentina |  |
|  |  |  |  | Chairperson: Barclay, Carlos |
|  |  |  |  |  |
| De Salvo, María C. MD | Gamberale, Ana M. MD | 186807/084932 | Centro Médico Dra De Salvo, Av. | Buenos Aires, Larrea 1381, |
|  | González Veliz, Angela M. MD |  | Cabildo 1548 1°A, Ciudad | Ciudad Autonoma de Buenos |
|  | Iannizzotto, Alejandra I. MD |  | Autónoma de Buenos Aires, | Aires, C1117ABK, Argentina |
|  | Luzardo Palacios, Vanessa M. |  | C1426ABO, Argentina |  |
|  | MD |  |  | Chairperson: Barclay, Carlos |
|  | Moreira Cedeña, Marcela M. |  |  |  |
|  | MD |  |  |  |
|  | Toro Briones, Rosana |  |  |  |
|  |  |  |  |  |

| **Investigator** | **Sub-Investigator** | **Investigator** | **Description of Research** | **Name of IEC/IRB Committee,** |
| --- | --- | --- | --- | --- |
|  |  | **no./Center no.** | **Facility, Hospital/ Institution,** | **Address, Committee Chair** |
|  |  |  | **and Address** |  |
|  |  |  |  |  |
| Defranchi, Hector A. | Marquez, Luis H. MD | 217573/083077 | Clinica del Sol., Av. Coronel Diaz | Buenos Aires, Larrea 1381, |
| MD |  |  | 2211, Ciudad Autonoma de | Ciudad Autonoma de Buenos |
|  |  |  | Buenos Aires, Buenos Aires, | Aires, C1117ABK, Argentina |
|  |  |  | 1425DQI, Argentina |  |
|  |  |  |  | Chairperson: Barclay, Carlos |
|  |  |  |  |  |
| Draghi, Jorge. MD | Giovanelli, Gustavo. MD | 238373/091433 | Hospital Regional Español Bahía | CER Instituto Médico, Vicente |
|  | Maruenda, Maximiliano. MD |  | Blanca, Estomba 571, Bahía | López 1441, Quilmes, Buenos |
|  | Mirofsky, Matías. MD |  | Blanca, Buenos Aires, | Aires, 1878, Argentina |
|  |  |  | B8000AAK, Argentina |  |
|  |  |  |  | Chairperson: Del Percio, Damian |
|  |  |  |  |  |
| Feola, Miguel A. MD | Casuso, Ana L | 235496/090636 | Instituto Medico Especializado, | Buenos Aires, Larrea 1381, |
|  | Lenge, Lorena. MD |  | Hidalgo 568, Ciudad Autonoma | Ciudad Autonoma de Buenos |
|  |  |  | de Buenos Aires, Buenos Aires, | Aires, C1117ABK, Argentina |
|  |  |  | C1405BCH, Argentina |  |
|  |  |  |  | Chairperson: Barclay, Carlos |
|  |  |  |  |  |

| **Investigator** | **Sub-Investigator** | **Investigator** | **Description of Research** | **Name of IEC/IRB Committee,** |
| --- | --- | --- | --- | --- |
|  |  | **no./Center no.** | **Facility, Hospital/ Institution,** | **Address, Committee Chair** |
|  |  |  | **and Address** |  |
|  |  |  |  |  |
| Garay, José E. MD | Alegre, Aníbal. MD | 238369/091431 | Hospital J.R. Vidal, c/Necochea | Buenos Aires, Larrea 1381, |
|  | González, Hugo R. MD |  | 1050, Corrientes, Corrientes, | Ciudad Autonoma de Buenos |
|  |  |  | W3410AVV, Argentina | Aires, C1117ABK, Argentina |
|  |  |  |  | Chairperson: Barclay, Carlos |
|  |  |  |  |  |
| Grilli, Monica B. MD | Fernandez, Ruben O. MD | 217566/083072 | Hospital Espanol, San Martin | Buenos Aires, Larrea 1381, |
|  |  |  | 965 Godoy Cruz, Godoy Cruz, | Ciudad Autonoma de Buenos |
|  |  |  | Mendoza, MQ 5500, Argentina | Aires, C1117ABK, Argentina |
|  |  |  |  | Chairperson: Barclay, Carlos |
|  |  |  |  |  |
| Homann, Sergio. MD | Becerra, Claudio C | 231835/090573 | Latino America Trials, San | Buenos Aires, Larrea 1381, |
|  | Ciruzzi, Domingo J |  | Martin 325 1 B, Cipolletti, Río | Ciudad Autonoma de Buenos |
|  |  |  | Negro, 8324, Argentina | Aires, C1117ABK, Argentina |
|  |  |  |  | Chairperson: Barclay, Carlos |
|  |  |  |  |  |

| **Investigator** | **Sub-Investigator** | **Investigator** | **Description of Research** | **Name of IEC/IRB Committee,** |
| --- | --- | --- | --- | --- |
|  |  | **no./Center no.** | **Facility, Hospital/ Institution,** | **Address, Committee Chair** |
|  |  |  | **and Address** |  |
|  |  |  |  |  |
| Lorenzati, Cristian D. | Alloati, Javier L. MD | 222474/085082 | Sanatorio Mayo S.A., Suipacha | Buenos Aires, Larrea 1381, |
| MD | Licheri, Alberto J. MD |  | 2453, Santa Fe, Santa Fe, | Ciudad Autonoma de Buenos |
| Rosselli, Rodolfo M. |  |  | S3000AZG, Argentina | Aires, C1117ABK, Argentina |
| (FPI) |  |  |  |  |
|  |  |  |  | Chairperson: Barclay, Carlos |
|  |  |  |  |  |
| Maillo, Martín Edgardo | Gattolin, Gabriel. MD | 234763/090572 | Instituto del Buen Aires, Junín | Buenos Aires, Larrea 1381, |
| Roberto. MD | Sgrignuoli, Carolina. MD |  | 2370, Santa Fe, Santa Fe, 3000, | Ciudad Autonoma de Buenos |
|  | Silva, Emiliana. MD |  | Argentina | Aires, C1117ABK, Argentina |
|  |  |  |  | Chairperson: Barclay, Carlos |
|  |  |  |  |  |
| Marzoratti, Lucia C. MD | Alvarez, Maria Soledad. MD | 187386/084934 | Centro Médico Investigadores | Buenos Aires, Larrea 1381, |
|  | Destefani Villafañe, Nicolas J. |  | Tucuman - CEMIT, Cordoba | Ciudad Autonoma de Buenos |
|  | MD |  | 256, Tucumán, Tucumán, | Aires, C1117ABK, Argentina |
|  | Figueroa, Sandra B. MD |  | T4000DGF, Argentina |  |
|  |  |  |  | Chairperson: Barclay, Carlos |
|  |  |  |  |  |
| Mattarucco, Walter J. | Aizenberg, Diego. MD | 224826/088394 | Centro Medico Viamonte, Avda. | Buenos Aires, Larrea 1381, |
| MD* | Glenny, Jorge A. MD |  | Cordoba 2019, Buenos Aires, | Ciudad Autonoma de Buenos |
|  |  |  |  |  |

| **Investigator** | **Sub-Investigator** | **Investigator** | **Description of Research** | **Name of IEC/IRB Committee,** |
| --- | --- | --- | --- | --- |
|  |  | **no./Center no.** | **Facility, Hospital/ Institution,** | **Address, Committee Chair** |
|  |  |  | **and Address** |  |
|  |  |  |  |  |
|  | Lo Giudice, Gustavo. MD |  | Buenos Aires, C1120AAC, | Aires, C1117ABK, Argentina |
|  | Navia, Edgardo G. MD |  | Argentina |  |
|  |  |  |  | Chairperson: Barclay, Carlos |
|  |  |  |  |  |
| Medina, Andrea C. MD | Pasquini, Marina G. MD | 217569/083075 | Centro de Investigaciones | CER Instituto Médico, Vicente |
|  | Real, Dafne L. MD |  | Medicas CIM, Carlos Galli | López 1441, Quilmes, Buenos |
|  |  |  | Mainini 192, Florencio Varela, | Aires, 1878, Argentina |
|  |  |  | 1888, Buenos Aires, Argentina |  |
|  |  |  |  | Chairperson: Del Percio, Damian |
|  |  |  |  |  |
| Medina, Iris V. MD | Bercovich, Javier G. MD | 066861/088388 | Centro Medico Vitae, Av. San | CER Instituto Médico, Vicente |
|  | Bertolot, German E. MD |  | Martin 966, 9 de Julio, Buenos | López 1441, Quilmes, Buenos |
|  | Cerutti, Lorena S. MD |  | Aires, B6500BWQ, Argentina | Aires, 1878, Argentina |
|  |  |  |  | Chairperson: Del Percio, Damian |
|  |  |  |  |  |
| Picone, Alejandro H. | Benítez, Sergio. MD | 238364/091511 | Sanatorio San Carlos, Lago | Buenos Aires, Larrea 1381, |
| MD | Freier, Gustavo. MD |  | Lacar Bustillo 15400 1, San | Ciudad Autonoma de Buenos |
|  |  |  | Carlos de Bariloche, Río Negro, | Aires, C1117ABK, Argentina |
|  |  |  | R8401DKA, Argentina |  |
|  |  |  |  |  |

| **Investigator** | **Sub-Investigator** | **Investigator** | **Description of Research** | **Name of IEC/IRB Committee,** |
| --- | --- | --- | --- | --- |
|  |  | **no./Center no.** | **Facility, Hospital/ Institution,** | **Address, Committee Chair** |
|  |  |  | **and Address** |  |
|  |  |  |  |  |
|  |  |  |  | Chairperson: Barclay, Carlos |
|  |  |  |  |  |
| Quiroga Barragán, | Aranda, Maria S. MD | 229951/088540 | CER San Juan, Laprida 568, | Buenos Aires, Larrea 1381, |
| Miguel E. MD | Castañeda, Francisco R. MD |  | San Juan, 5400, Argentina | Ciudad Autonoma de Buenos |
|  | Castro, Luis A. MD |  |  | Aires, C1117ABK, Argentina |
|  | Gregorini, Rosanna V. MD |  |  |  |
|  | Lanzone, Silvana. MD |  |  | Chairperson: Barclay, Carlos |
|  | Vellio, María A. MD |  |  |  |
|  |  |  |  |  |
| Raso, Ernesto R. MD | Calella, Pedro. MD | 229648/088538 | CIDAPI, Libertad 2236, San | Buenos Aires, Larrea 1381, |
|  |  |  | Rafael, Mendoza, M5602HWT , | Ciudad Autonoma de Buenos |
|  |  |  | Argentina | Aires, C1117ABK, Argentina |
|  |  |  |  | Chairperson: Barclay, Carlos |
|  |  |  |  |  |

| **Investigator** | **Sub-Investigator** | **Investigator** | **Description of Research** | **Name of IEC/IRB Committee,** |  |
| --- | --- | --- | --- | --- | --- |
|  |  | **no./Center no.** | **Facility, Hospital/ Institution,** | **Address, Committee Chair** |  |
|  |  |  | **and Address** |  |  |
|  |  |  |  |  |  |
| Rodriguez, Pablo O. | Mas, Juan M. MD | 217567/083074 | CEMIC, Av Galvan 4102, Ciudad | CEMIC, Galvan 21, Buenos |  |
| MD | Maskin, Patricio. MD |  | Autonoma de Buenos Aires, | Aires, Buenos Aires, Argentina |  |
|  |  |  | Buenos Aires, C1431FWO, | Chairperson: Krupitzki, Hugo |  |
|  |  |  | Argentina |  |  |
|  |  |  |  |  |  |
|  |  |  |  |  |  |
| Taborda, Jorge. MD | Alvarisqueta, Andres. MD | 234536/091432 | Clinica de Fracturas y | CER Instituto Médico, Vicente |  |
|  | Ramonda, Andrea. MD |  | Ortopedia, Av.Independencia | López 1441, Quilmes, , Buenos |  |
|  | Reig, Moira. MD |  | 1475, Mar del Plata, Buenos | Aires, 1878, Argentina |  |
|  | Suarez, Gabriel. MD |  | Aires, 7600, Argentina | Chairperson: Del Percio, Damian |  |
|  |  |  |  |  |  |
|  |  |  |  |  |  |
| Victorio, Carlos F. MD | O´Farrell, María L. MD | 231908/091430 | Centro de Medicina Respiratoria, | Buenos Aires, Larrea 1381, |  |
|  |  |  | Peru 132, Concepcion del | Ciudad Autonoma de Buenos |  |
|  |  |  | Uruguay, Entre Ríos, | Aires, C1117ABK, Argentina |  |
|  |  |  | E3260EPD, Argentina | Chairperson: Barclay, Carlos |  |
|  |  |  |  |  |  |
|  |  |  |  |  |  |
| Wehbe, Luis A. MD | Casero, Fernando M. MD | 144351/084929 | Ave Pulmo, Carlos M. Alvear | CER Instituto Médico, Vicente |  |
|  | De La vega, Carlos M. MD |  | 3345, Mar del Plata, Buenos | López 1441, Quilmes, Buenos |  |
|  | Sabas, Mario F. MD |  | Aires, 7600, Argentina | Aires, 1878, Argentina |  |
|  |  |  |  | Chairperson: Del Percio, Damian |  |
|  |  |  |  |  |  |

| **Investigator** | **Sub-Investigator** | **Investigator** | **Description of Research** | **Name of IEC/IRB Committee,** |
| --- | --- | --- | --- | --- |
|  |  | **no./Center no.** | **Facility, Hospital/ Institution,** | **Address, Committee Chair** |
|  |  |  | **and Address** |  |
|  |  |  |  |  |
| **Norway** |  |  |  |  |
|  |  |  |  |  |
| Eskeland, Inge. MD | Aasen, Odd Frode | 002111/086543 | Helse Sunnmøre HF. Volda | Regional komite for medisinsk og |
|  |  |  | Sjukehus, Kløvertunveien 1, | helsefaglig forskningsetikk, REK |
|  |  |  | Volda, 6100, Norway | midt, Det medisinske fakultet, |
|  |  |  |  | Medisinsk teknisk |
|  |  |  |  | forskningssente, 7489, |
|  |  |  |  | Trondheim, Norway. |
|  |  |  |  | Chairperson: Gisvold, Sven E |
|  |  |  |  |  |
| Galaaen, Bent. MD | Berg, Janna | 221027/084474 | Sykehuset i Vestfold HF, | Regional komite for medisinsk og |
|  |  |  | Tønsberg, Lungeseksjonen, | helsefaglig forskningsetikk, REK |
|  |  |  | Halfdan Wilhelmsens alle 17, | midt, Det medisinske fakultet, |
|  |  |  | Tønsberg, 3116, Norway | Medisinsk teknisk |
|  |  |  |  | forskningssenter 7489, |
|  |  |  |  | Trondheim, Norway. |
|  |  |  |  | Chairperson: Gisvold, Sven E |
|  |  |  |  |  |

| **Investigator** | **Sub-Investigator** | **Investigator** | **Description of Research** | **Name of IEC/IRB Committee,** |
| --- | --- | --- | --- | --- |
|  |  | **no./Center no.** | **Facility, Hospital/ Institution,** | **Address, Committee Chair** |
|  |  |  | **and Address** |  |
|  |  |  |  |  |
| Naustdal, Thor. MD | Kibsgaard, Regina | 214140/083739 | Heise Nord-Trondelag HF, | Regional komite for medisinsk og |
|  | Oppegaard, Per |  | Sykehuset Levanger, Kirkegaten | helsefaglig forskningsetikk, REK |
|  |  |  | 2, Levanger, 7600, Norway | midt, Det medisinske fakultet, |
|  |  |  |  | Medisinsk teknisk |
|  |  |  |  | forskningssenter 7489, |
|  |  |  |  | Trondheim, Norway. |
|  |  |  |  | Chairperson: Gisvold, Sven E |
|  |  |  |  |  |
| Saleh, Safaa. MBChB | Madebo, Tesfaye | 221774/084815 | Stavanger Universitetssjukehus, | Regional komite for medisinsk og |
|  | Reite, Mads |  | Armauer Hansens vei 20, | helsefaglig forskningsetikk, REK |
|  |  |  | Stavanger\|, 4068, Norway | midt, Det medisinske fakultet, |
|  |  |  |  | Medisinsk teknisk |
|  |  |  |  | forskningssenter 7489, |
|  |  |  |  | Trondheim, Norway. |
|  |  |  |  | Chairperson: Gisvold, Sven E |
|  |  |  |  |  |

| **Investigator** | **Sub-Investigator** | **Investigator** | **Description of Research** | **Name of IEC/IRB Committee,** |
| --- | --- | --- | --- | --- |
|  |  | **no./Center no.** | **Facility, Hospital/ Institution,** | **Address, Committee Chair** |
|  |  |  | **and Address** |  |
|  |  |  |  |  |
| Sue-Chu, Malcolm. | Brønstad, Eivind | 001949/083852 | St. Olavs Hospital HF., Seksjon | Regional komite for medisinsk og |
| MBChB, PhD, FRCA* |  |  | for lungesykdommer, Prinsesse | helsefaglig forskningsetikk, REK |
|  |  |  | Kristinasgt. 3, Trondheim, 7030, | midt, Det medisinske fakultet, |
|  |  |  | Norway | Medisinsk teknisk |
|  |  |  |  | forskningssenter 7489, |
|  |  |  |  | Trondheim, Norway. |
|  |  |  |  | Chairperson: Gisvold, Sven E |
|  |  |  |  |  |
| Tollåli, Terje. MD* | Flatøy, Liv R. | 002144/084420 | Nordlandssykehuset HF, | Regional komite for medisinsk og |
|  |  |  | Med.avd., Prinsensgate 164, | helsefaglig forskningsetikk, REK |
|  |  |  | Bodø, 8005, Norway | midt, Det medisinske fakultet, |
|  |  |  |  | Medisinsk teknisk |
|  |  |  |  | forskningssenter 7489, |
|  |  |  |  | Trondheim, Norway. |
|  |  |  |  | Chairperson: Gisvold, Sven E |
|  |  |  |  |  |

| **Investigator** | **Sub-Investigator** | **Investigator** | **Description of Research** | **Name of IEC/IRB Committee,** |
| --- | --- | --- | --- | --- |
|  |  | **no./Center no.** | **Facility, Hospital/ Institution,** | **Address, Committee Chair** |
|  |  |  | **and Address** |  |
|  |  |  |  |  |
| **United States** |  |  |  |  |
|  |  |  |  |  |
| Abboy, Chandar. MD* | Bradberry, Jenny P. BS (CRC) | 194755/076839 | Greenville Pharmaceutical | Quorum Review, Inc., Suite |
|  | Clemons, Jonnalee. CCRC |  | Research, Suite B, 220 Roper | 1000, 1601 Fifth Avenue, |
|  | Clemons, Steven L. MSN, |  | Mountain Road Extension, | Seattle, Washington, 98101, |
|  | FNP, CCRC |  | Greenville, South Carolina, | United States. |
|  | Freeman III, Carlos A. AS, |  | 29615, United States |  |
|  | CRT, RCP (CRC) |  |  | Chairperson: Kelley, David |
|  | Peay, Kayla N. BS (CRC) |  |  |  |
|  | Tate, Tammy L. CNA, CCRC |  |  |  |
|  | Tyson, Stefanie N. AS, CCRC |  |  |  |
|  |  |  |  |  |
| Boeren, John J .MD* | Capasso-Gulve, Elizabeth. PA- | 031413/075268 | Belleville Family Medical | Quorum Review, Inc., Suite |
|  | C |  | Associates, Ltd., Suite 300, 311 | 1000, 1601 Fifth Avenue, |
|  | Wolff III, Edward A. MD |  | West Lincoln Street, Belleville, | Seattle, Washington, 98101, |
|  | Wood, Kelly A. MD |  | Illinois, 62220, United States | United States. |
|  |  |  |  | Chairperson: Kelley, David |
|  |  |  |  |  |

| **Investigator** | **Sub-Investigator** | **Investigator** | **Description of Research** | **Name of IEC/IRB Committee,** |
| --- | --- | --- | --- | --- |
|  |  | **no./Center no.** | **Facility, Hospital/ Institution,** | **Address, Committee Chair** |
|  |  |  | **and Address** |  |
|  |  |  |  |  |
| Boscia, III, Joseph A. | Benfield, Meredith. RRT, | 067189/073902 | CU Pharmaceutical Research, | Quorum Review, Inc., Suite |
| MD, CPI | CCRC |  | 1005 Thompson Blvd., Union, | 1000, 1601 Fifth Avenue, |
|  | Buice, J. Nicole. RN, CCRC |  | South Carolina, 29379, United | Seattle, Washington, 98101, |
|  | DeLaCruz, Luis I. MD |  | States | United States. |
|  | Duncan, Bonnie. RTR, CRC |  |  |  |
|  | Feldman, Gregory J. MD |  |  | Chairperson: Kelley, David |
|  | Gossett, Marsha. CRC |  |  |  |
|  | Raynor, Anthony. PA-C |  |  |  |
|  | Shetley, Tara. CRC |  |  |  |
|  | Sprinkle, Brenda. PA-C |  |  |  |
|  | Stockinger, Helen. MD |  |  |  |
|  | Todd, Mary Elizabeth. CRC |  |  |  |
|  | Williams, Haley. RRT, CCRC |  |  |  |
|  | Witt, Amy. CST, CRC |  |  |  |
|  |  |  |  |  |

| **Investigator** | **Sub-Investigator** | **Investigator** | **Description of Research** | **Name of IEC/IRB Committee,** |
| --- | --- | --- | --- | --- |
|  |  | **no./Center no.** | **Facility, Hospital/ Institution,** | **Address, Committee Chair** |
|  |  |  | **and Address** |  |
|  |  |  |  |  |
| Bruya, Timothy E. MD* | Banasik, Jacquelyn L. ARNP | 009865/074058 | Pulmonary & Research | Quorum Review, Inc., Suite |
|  | Bruya, Margaret |  | Associates, Suite 400, 104 West | 1000, 1601 Fifth Avenue, |
|  | Gower, Richard G. MD |  | 5th Avenue, Spokane, | Seattle, Washington, 98101, |
|  | Levitch, E. Suzanne. ARNP |  | Washington, 99204, United | United States. |
|  | Loewen, Gregory. DO |  | States |  |
|  |  |  |  | Chairperson: Kelley, David |
|  |  |  |  |  |
| Chandran, Ravi. MD | Hayden, Angela C. APRN | 170417/075274 | HOPE Pharmaceutical | Quorum Review, Inc., Suite |
|  |  |  | Research, 959 Bypass 123, | 1000, 1601 Fifth Avenue, |
|  |  |  | Suites C and D, Seneca, South | Seattle, Washington, 98101, |
|  |  |  | Carolina, 29678, United States. | United States. |
|  |  |  |  | Chairperson: Kelley, David |
|  |  |  |  |  |
| Cooper, John Allen | Dransfield, Mark. MD | 009688/075225 | Birmingham VAMC Pulmonary | Birmingham VA Medical Center, |
| Dicks Jr. MD |  |  | Rooms 3333, 3334 3305, 700 | Research Services, 700 South |
|  |  |  | South 19 Street, Birmingham, | 19th Street, Birmingham, |
|  |  |  | Alabama, 35233, United States | Alabama, 35233, United States |
|  |  |  |  |  |

| **Investigator** | **Sub-Investigator** | **Investigator** | **Description of Research** | **Name of IEC/IRB Committee,** |  |
| --- | --- | --- | --- | --- | --- |
|  |  | **no./Center no.** | **Facility, Hospital/ Institution,** | **Address, Committee Chair** |  |
|  |  |  | **and Address** |  |  |
|  |  |  |  |  |  |
|  |  |  |  | Chairperson: Parker, Pamela |  |
|  |  |  |  |  |  |
| Criner, Gerard. MD* | Chatila, Wissam. MD | 029275/074059 | Temple University Hospital, 7th | Western Internatinal Review |  |
|  | Mamary, Albert J. MD |  | Floor, Parkinson Pavilion, 3401 | Board, 3535 Seventh Avenue, |  |
|  | Marchetti, Nathaniel. DO |  | North Broad Street, Philadelphia, | SW, Olympia, Washington, |  |
|  | Satti, Aditi G. MD |  | Pennsylvania, 19140, United | 98502, United States |  |
|  |  |  | States | Chairperson: Schultz, Theodore |  |
|  |  |  |  |  |  |
|  |  |  |  |  |  |
| Cruz, Humberto. DO* | None | 175852/075272 | Florida Institue for Clinical | Quorum Review, Inc., Suite |  |
|  |  |  | Research, 7200 Curry Ford | 1000, 1601 Fifth Avenue, |  |
|  |  |  | Road, Orlando, Florida, 32822, | Seattle, Washington, 98101, |  |
|  |  |  | United States | United States. |  |
|  |  |  |  | Chairperson: Kelley, David |  |
|  |  |  |  |  |  |
| Daniel, Saifu. MD | Buck, Theresa. MD | 015156/073951 | Bay Pines VA Medical Center, | Human Studies Subcommittee, |  |
| Peniston, Reginald L. | Lamendola, Lynnette. ARNP |  | Bldg 100 Rm 5D-151, 10000 Bay | Bay Pines VA Healthcare |  |
| MD (FPI) |  |  | Pines Blvd, Bay Pines, Florida, | System, Research and |  |
|  |  |  | 33744, United States | Development Service (151), |  |
|  |  |  |  | 10000 Bay Pines Blvd, Bay |  |
|  |  |  |  |  |  |

| **Investigator** | **Sub-Investigator** | **Investigator** | **Description of Research** | **Name of IEC/IRB Committee,** |
| --- | --- | --- | --- | --- |
|  |  | **no./Center no.** | **Facility, Hospital/ Institution,** | **Address, Committee Chair** |
|  |  |  | **and Address** |  |
|  |  |  |  |  |
|  |  |  |  | Pines, 33744, Florida, United |
|  |  |  |  | States |
|  |  |  |  | Chairperson: Langhans, Joseph |
|  |  |  |  |  |
| Delaney, Morgan* | Desrosiers, Aimee | 177335/073953 | George Washington University | Western Internatinal Review |
|  | Hasselquist, Susan |  | Medical Center, 2150 | Board, 3535 Seventh Avenue, |
|  |  |  | Pennsylvania Avenue, NW, | SW, Olympia, Washington, |
|  |  |  | Washington, District of | 98502, United States |
|  |  |  | Columbia, 20037, United States |  |
|  |  |  |  | Chairperson: Schultz, Theodore |
|  |  |  |  |  |
| Diener, Carl F. MD* | Patton, Walter S. MD, | 105931/074374 | Verona Clinical Research, Suite | Quorum Review, Inc., Suite |
|  | FACOG, CPI |  | 285, 6565 E. Carondelet Drive, | 1000, 1601 Fifth Avenue, |
|  |  |  | Tucson, Arizona, 85710, United | Seattle, Washington, 98101, |
|  |  |  | States | United States. |
|  |  |  |  | Chairperson: Kelley, David |
|  |  |  |  |  |

| **Investigator** | **Sub-Investigator** | **Investigator** | **Description of Research** | **Name of IEC/IRB Committee,** |
| --- | --- | --- | --- | --- |
|  |  | **no./Center no.** | **Facility, Hospital/ Institution,** | **Address, Committee Chair** |
|  |  |  | **and Address** |  |
|  |  |  |  |  |
| Dransfield, Mark T. MD | Kirkpatrick, deNay. MSN, | 012441/074060 | UAB Lung Health Center, 526 | Western Internatinal Review |
|  | CRNP |  | 20th Street South, Birmingham, | Board, 3535 Seventh Avenue, |
|  | Wells, James M .MD |  | Alabama, 25294, United States | SW, Olympia, Washington, |
|  |  |  |  | 98502, United States |
|  |  |  |  | Chairperson: Schultz, Theodore |
|  |  |  |  |  |
| El Bayadi, Sherif G | Agne, Jill. FNP | 190902/075278 | Medical Research Associates of | Quorum Review, Inc., Suite |
|  | Keenan, Deirdre. RPA-C |  | Central New York, PLLC, 5229 | 1000, 1601 Fifth Avenue, |
|  | Schaeffer, Ellen. MD |  | Witz Drive, North Syracuse, New | Seattle, Washington, 98101, |
|  | Sotomayor, Juan L. MD |  | York, 13212, United States | United States. |
|  |  |  |  | Chairperson: Kelley, David |
|  |  |  |  |  |

| **Investigator** | **Sub-Investigator** | **Investigator** | **Description of Research** | **Name of IEC/IRB Committee,** |
| --- | --- | --- | --- | --- |
|  |  | **no./Center no.** | **Facility, Hospital/ Institution,** | **Address, Committee Chair** |
|  |  |  | **and Address** |  |
|  |  |  |  |  |
| Erb, David R. MD | Abboy, Chandar. MD | 067190/073957 | Gaffney Pharmaceutical | Quorum Review, Inc., Suite |
|  | Boscia, III, Joseph A. MD |  | Research, 1529 North Limestone | 1000, 1601 Fifth Avenue, |
|  | Dela Cruz, Luis. MD |  | Street, Gaffney, South Carolina, | Seattle, Washington, 98101, |
|  | Erwin, Ashley D. |  | 29340, United States | United States. |
|  | Esposito, Kendall M |  |  |  |
|  | Feldman, Gregory J. MD |  |  | Chairperson: Kelley, David |
|  | Morris, Janice P |  |  |  |
|  | White, Kelly N |  |  |  |
|  |  |  |  |  |
| Fakih, Faisal A. MD | Gmerek-Pettus, Sabrina. | 113086/074116 | Florida Pulmonary Research | Quorum Review, Inc., Suite |
|  | ARNP |  | Center, Suite B, 1788 West | 1000, 1601 Fifth Avenue, |
|  |  |  | Fairbanks Avenue, Winter Park,, | Seattle, Washington, 98101, |
|  |  |  | Florida, 32789, United States | United States. |
|  |  |  |  | Chairperson: Kelley, David |
|  |  |  |  |  |

| **Investigator** | **Sub-Investigator** | **Investigator** | **Description of Research** | **Name of IEC/IRB Committee,** |
| --- | --- | --- | --- | --- |
|  |  | **no./Center no.** | **Facility, Hospital/ Institution,** | **Address, Committee Chair** |
|  |  |  | **and Address** |  |
|  |  |  |  |  |
| Feldman, Gregory J. | Allison, Heather. CPhT, BBA, | 061057/073959 | South Carolina Pharmaceutical | Quorum Review, Inc., Suite |
| MD | CCRC |  | Research, Suite 2100, 1330 | 1000, 1601 Fifth Avenue, |
|  | Arrellaw, Maritza |  | Boiling Springs Road, | Seattle, Washington, 98101, |
|  | Ayers, Allison N |  | Spartanburg, South Carolina, | United States. |
|  | Boscia, III, Joseph A. MD, |  | 29303, United States |  |
|  | FCCP, CPI |  |  | Chairperson: Kelley, David |
|  | Buice, Anna M |  |  |  |
|  | Cannon, Kimberly B |  |  |  |
|  | Carson, Heather N |  |  |  |
|  | Chapman, Candice E |  |  |  |
|  | Collins, Emily C. RN, BSN |  |  |  |
|  | Jolly, Laura R |  |  |  |
|  | Leopard, Tammy |  |  |  |
|  | Lyda, Krysti N |  |  |  |
|  | Pittman, Tammy T. LPN |  |  |  |
|  | Raynor, Anthony W. PA-C |  |  |  |
|  | Taylor, Wendy |  |  |  |
|  |  |  |  |  |

| **Investigator** | **Sub-Investigator** | **Investigator** | **Description of Research** | **Name of IEC/IRB Committee,** |
| --- | --- | --- | --- | --- |
|  |  | **no./Center no.** | **Facility, Hospital/ Institution,** | **Address, Committee Chair** |
|  |  |  | **and Address** |  |
|  |  |  |  |  |
| Ferguson, Gary T. MD | None | 015203/073961 | Pulmonary Research Institute of | Botsford General Hospital, |
|  |  |  | Southeast Michigan, Suite 103, | 28050 Grand River Ave,, |
|  |  |  | 28815 Eight Mile Road, Livonia, | Farmington Hills, MJ 48336- |
|  |  |  | Michigan, 48152, United States | 5919. United States |
|  |  |  |  | Chairperson: Powell, Vance |
|  |  |  |  |  |
| Fiedler, Douglas R. | Chakraborty, Anup K. MD | 176321/073962 | Nebraska Pulmonary Specialties | Quorum Review, Inc., Suite |
| MD* | Jarrett, Jeffrey E. MD |  | LLC, 1500 S 48th Street, Lincoln, | 1000, 1601 Fifth Avenue, |
|  | Johnson, William M. MD |  | Nebraska, 68506, United States | Seattle, Washington, 98101, |
|  | Kleinschmidt, Carrie. PA-C |  |  | United States |
|  | Mansur, Lisa I. MD |  |  |  |
|  | Miller, Ellen G. MD |  |  | Chairperson: Kelley, David |
|  | Reichmuth, Kevin J. MD |  |  |  |
|  | Rudersdorf, John H. MD |  |  |  |
|  | Sorensen, Scott. PA-C |  |  |  |
|  | Trapp, John F. MD |  |  |  |
|  |  |  |  |  |

| **Investigator** | **Sub-Investigator** | **Investigator** | **Description of Research** | **Name of IEC/IRB Committee,** |
| --- | --- | --- | --- | --- |
|  |  | **no./Center no.** | **Facility, Hospital/ Institution,** | **Address, Committee Chair** |
|  |  |  | **and Address** |  |
|  |  |  |  |  |
| Giessel, Glenn M. MD | Hey, Jamie C. MD | 021992/073966 | Pulmonary Associates of | Quorum Review, Inc., Suite |
|  | Jones IV, Drew G. MD |  | Richmond, Inc., 1000 Boulders | 1000, 1601 Fifth Avenue, |
|  | Kuno, Ritsu. MD |  | Parkway- Suite 201, Richmond, | Seattle, Washington, 98101, |
|  | Mathers Jr., James A. MD |  | Virginia, 23225, United States | United States |
|  | Polsky, Michael B. MD |  |  |  |
|  | Rivers, Cullen B. MD |  |  | Chairperson: Kelley, David |
|  | Sarna, Pawanjit S. MD |  |  |  |
|  | Smith, Rodney H. MD |  |  |  |
|  | Taylor, Cynthia A. ACNP |  |  |  |
|  | Torrisi, Peter F. MD |  |  |  |
|  |  |  |  |  |
| Given, John T. MD | Moyer, Jennifer M. CNP | 018832/074065 | Allergy and Respiratory Center, | Quorum Review, Inc., Suite |
|  |  |  | 4048 Dressler Road NW, | 1000, 1601 Fifth Avenue, |
|  |  |  | Canton, Ohio, 44718, United | Seattle, Washington, 98101, |
|  |  |  | States | United States |
|  |  |  |  | Chairperson: Kelley, David |
|  |  |  |  |  |

| **Investigator** | **Sub-Investigator** | **Investigator** | **Description of Research** | **Name of IEC/IRB Committee,** |
| --- | --- | --- | --- | --- |
|  |  | **no./Center no.** | **Facility, Hospital/ Institution,** | **Address, Committee Chair** |
|  |  |  | **and Address** |  |
|  |  |  |  |  |
| Gotfried, Mark H. MD | Anthony, Stephen R .MD | 010875/074118 | Pulmonary Associates PA, 1112 | Quorum Review, Inc., Suite |
|  | Comp, Robert A. MD |  | East McDowell Road, Phoenix, | 1000, 1601 Fifth Avenue, |
|  | Gourdoux, Stacey A. ANP-C |  | Arizona, 85006, United States | Seattle, Washington, 98101, |
|  | Grill, Jeffrey A. MD |  |  | United States |
|  | Heaphy, Lynda S. FNP-C |  |  |  |
|  | Newell, Judith N. CFNP |  |  | Chairperson: Kelley, David |
|  | Ramakrishna, Sripathi. MD |  |  |  |
|  | Ross, J Burr. MD |  |  |  |
|  |  |  |  |  |
| Graif, Joseph. MD* | Hanovich, Steven. MD | 198695/074703 | Cities Lung Clinic, Suite 360, 500 | Quorum Review, Inc., Suite |
|  |  |  | Osborne Road, Fridley, | 1000, 1601 Fifth Avenue, |
|  |  |  | Minnesota, 55432, United States | Seattle, Washington, 98101, |
|  |  |  |  | United States |
|  |  |  |  | Chairperson: Kelley, David |
|  |  |  |  |  |

| **Investigator** | **Sub-Investigator** | **Investigator** | **Description of Research** | **Name of IEC/IRB Committee,** |
| --- | --- | --- | --- | --- |
|  |  | **no./Center no.** | **Facility, Hospital/ Institution,** | **Address, Committee Chair** |
|  |  |  | **and Address** |  |
|  |  |  |  |  |
| Hales, Jeff B. MD* | Glasser, David. PA-C | 176315/086467 | Clinical Research & Consulting | Quorum Review, Inc., Suite |
|  | Jacobson, Michael. MSHS, PA- |  | Center, LLC, Suite 101, 3930 | 1000, 1601 Fifth Avenue, |
|  | C |  | Walnut St., Fairfax, Virginia, | Seattle, Washington, 98101, |
|  | Wyckoff, Christopher. MD |  | 22030, United States | United States |
|  | Zimmet, Steven. MD |  |  |  |
|  |  |  |  | Chairperson: Kelley, David |
|  |  |  |  |  |
| Heyder, Albrecht | None | 017815/073970 | Carolina Research Specialists, | Quorum Review, Inc., Suite |
|  |  |  | 111- A Medical Drive, Elizabeth | 1000, 1601 Fifth Avenue, |
|  |  |  | City, North Carolina, 27909, | Seattle, Washington, 98101, |
|  |  |  | United States | United States |
|  |  |  |  | Chairperson: Kelley, David |
|  |  |  |  |  |
| Hoffman, Kent S. DO | Samano III, Gregory P. DO | 022676/073973 | Alliance Clinical Research, 2830 | Quorum Review, Inc., Suite |
|  |  |  | Casa Aloma Way, Winter Park, | 1000, 1601 Fifth Avenue, |
|  |  |  | Florida, 32792, United States | Seattle, Washington, 98101, |
|  |  |  |  | United States |
|  |  |  |  | Chairperson: Kelley, David |
|  |  |  |  |  |

| **Investigator** | **Sub-Investigator** | **Investigator** | **Description of Research** | **Name of IEC/IRB Committee,** |
| --- | --- | --- | --- | --- |
|  |  | **no./Center no.** | **Facility, Hospital/ Institution,** | **Address, Committee Chair** |
|  |  |  | **and Address** |  |
|  |  |  |  |  |
| Hyers, Thomas M. MD* | Jackson, Jr., Farris. MD | 016377/074070 | CARE Clinical Research, Suite | Quorum Review, Inc., Suite |
|  |  |  | 350, 522 North New Ballas | 1000, 1601 Fifth Avenue, |
|  |  |  | Road, St. Louis, Missouri, 63141, | Seattle, Washington, 98101, |
|  |  |  | United States | United States |
|  |  |  |  | Chairperson: Kelley, David |
|  |  |  |  |  |
| Joseph, Jose. MD* | Escobar, Andres. MD | 174200/074072 | University Central Medical | Community Medical Centers |
|  |  |  | Specialty Clinic, 2828 Fresno | IRB, Department of Internal |
|  |  |  | Street, Fresno, California, 93721, | Medicine, University Medical |
|  |  |  | United States | Center, 445 S. Cedar Avenue, |
|  |  |  |  | Fresno, California, 93702, United |
|  |  |  |  | States |
|  |  |  |  | Chairperson Libke, Robert |
|  |  |  |  |  |

| **Investigator** | **Sub-Investigator** | **Investigator** | **Description of Research** | **Name of IEC/IRB Committee,** |
| --- | --- | --- | --- | --- |
|  |  | **no./Center no.** | **Facility, Hospital/ Institution,** | **Address, Committee Chair** |
|  |  |  | **and Address** |  |
|  |  |  |  |  |
| Kaelin, Jr., Thomas D. | DesNoyer, Erin. PA-C | 017720/073974 | LowCountry Lung & Critical | Quorum Review, Inc., Suite |
| DO | Handshoe, David K. MD |  | Care, P.A., 9150 B Medcom | 1000, 1601 Fifth Avenue, |
|  | Miller, K. Scott. MD |  | Street, Charleston, South | Seattle, Washington, 98101, |
|  | Rucker, John M. MD |  | Carolina, 29406-7108, United | United States |
|  |  |  | States |  |
|  |  |  |  | Chairperson: Kelley, David |
|  |  |  |  |  |
| Kaye, Mitchell. MD | Bowen, Robert M. MD | 015503/074074 | Minnesota Lung Center, Suite | Quorum Review, Inc., Suite |
|  | Doepke, Matt. CRC, CphT, |  | 700, 920 East 28th Street, | 1000, 1601 Fifth Avenue, |
|  | PBT |  | Minneapolis, Minnesota, 55407, | Seattle, Washington, 98101, |
|  | Flashinski, Doug. MN, RN, |  | United States | United States |
|  | CRC |  |  |  |
|  | Hawthorne, April L. RN, CRC |  |  | Chairperson: Kelley, David |
|  | Peterson, Erin. NP-C |  |  |  |
|  | Stern, Wayne. MD |  |  |  |
|  | Udovich, Patricia. NP-C |  |  |  |
|  | Walters, Mallori. CRC, PBT |  |  |  |
|  |  |  |  |  |

| **Investigator** | **Sub-Investigator** | **Investigator** | **Description of Research** | **Name of IEC/IRB Committee,** |
| --- | --- | --- | --- | --- |
|  |  | **no./Center no.** | **Facility, Hospital/ Institution,** | **Address, Committee Chair** |
|  |  |  | **and Address** |  |
|  |  |  |  |  |
| Klein, Ryan M. MD, | Marks, Melvin I. MD | 225043/086447 | Huntington Beach Internal | Quorum Review, Inc., Suite |
| FCCP | Rosenblatt, Stanley. MD |  | Medicine Group, Inc., Suite 111, | 1000, 1601 Fifth Avenue, |
|  |  |  | 1501 Superior Ave, Newport | Seattle, Washington, 98101, |
|  |  |  | Beach, California, 92663, United | United States |
|  |  |  | States |  |
|  |  |  |  | Chairperson: Kelley, David |
|  |  |  |  |  |
| Koser, Andras. MD, | Clemons, Gary L. RN, CCRC | 074855/073976 | Palmetto Medical Research | Quorum Review, Inc., Suite |
| FHM, MBA, CCPI | Clemons, Johnalee. CCRC |  | Associates, 201 South B Street, | 1000, 1601 Fifth Avenue, |
|  | Clemons, Melissa M. BS, PTA |  | Easley, South Carolina, 29640, | Seattle, Washington, 98101, |
|  | (CRC) |  | United States | United States |
|  | Clemons, Steven L. MSN, |  |  |  |
|  | FNP, CCRC |  |  | Chairperson: Kelley, David |
|  | Herrold, Tara Mullinax. BS, |  |  |  |
|  | CRC |  |  |  |
|  | Holley, Jacob G. BS, CRC |  |  |  |
|  | Lazariashvili, Nikoloz. MD |  |  |  |
|  |  |  |  |  |

| **Investigator** | **Sub-Investigator** | **Investigator** | **Description of Research** | **Name of IEC/IRB Committee,** |  |
| --- | --- | --- | --- | --- | --- |
|  |  | **no./Center no.** | **Facility, Hospital/ Institution,** | **Address, Committee Chair** |  |
|  |  |  | **and Address** |  |  |
|  |  |  |  |  |  |
| Laman, P. David. MD | Deliere, Emil. MD | 009686/074112 | Pittsburgh Pulmonary | Quorum Review, Inc., Suite |  |
|  | Madison, Jan. MD |  | Associates, JMA Building, 1200 | 1000, 1601 Fifth Avenue, |  |
|  | Puri, Ankur. MD |  | Brooks Lane, Suite 130, | Seattle, Washington, 98101, |  |
|  | Shuman, Tobi. MD |  | Pittsburgh, Pennsylvania, 15025, | United States |  |
|  | Sims, William. MD |  | United States | Chairperson: Kelley, David |  |
|  |  |  |  |  |  |
|  |  |  |  |  |  |
| Lee, Mitchell D. MD | Cannon, Kevin D. MD | 020449/074077 | PMG Research of Wilmington | Quorum Review, Inc., Suite |  |
|  | Deeley, Beth A. PA-C |  | LLC, 1202 Medical Center Drive, | 1000, 1601 Fifth Avenue, |  |
|  |  |  | Wilmington, North Carolina, | Seattle, Washington, 98101, |  |
|  |  |  | 28401, United States | United States |  |
|  |  |  |  | Chairperson: Kelley, David |  |
|  |  |  |  |  |  |
| Levin, David C. MD* | Ellis, Ashley. RN, BSN | 009147/075231 | VA Medical Center, Oklahoma | The University Of Oklahoma |  |
|  | Jones, Kellie R. MD |  | City, 921 North East 13th Street, | Health Science Center, LIB121, |  |
|  |  |  | Oklahoma City, Oklahoma, | 1000 Stanton L. Young Blvd., |  |
|  |  |  | 73104, United States | Oklahoma City, 73117, United |  |
|  |  |  |  | States |  |
|  |  |  |  | Chairperson: Jelley, Martina |  |
|  |  |  |  |  |  |

| **Investigator** | **Sub-Investigator** | **Investigator** | **Description of Research** | **Name of IEC/IRB Committee,** |
| --- | --- | --- | --- | --- |
|  |  | **no./Center no.** | **Facility, Hospital/ Institution,** | **Address, Committee Chair** |
|  |  |  | **and Address** |  |
|  |  |  |  |  |
| Levinson, Gary D. MD* | Hartwich, Martha C. NP | 011096/074599 | Integrated Research Center, | Quorum Review, Inc., Suite |
|  | Kidokoro, Yasuko. MD |  | Suite 303, 4282 Genesee Ave, | 1000, 1601 Fifth Avenue, |
|  | Laliotis, Aristotelis T. MD |  | San Diego, California, 92117, | Seattle, Washington, 98101, |
|  |  |  | United States | United States. |
|  |  |  |  | Chairperson: Kelley, David |
| Liu, Jing. MD | Hassan, Zubair. MD | 022501/074115 | Robert J Dole VA Medical | Kansas City Veterans |
|  |  |  | Center, 11SC, 5500 E Kellogg | Administration Medical Center, |
|  |  |  | (SC), Wichita, Kansas, 67218, | Research Building 15, Rm 121, |
|  |  |  | United States | 4801 Linwood Blvd, Kansas City, |
|  |  |  |  | Missouri, 64128, United States |
|  |  |  |  | Chairperson: Sheedy, Carmen |
| Liu, Mark C. MD | Hansell, Nadia. MD | 013976/073980 | John Hopkins University, | Johns Hopkins Institutional |
|  |  |  | Baltimore, 1B-76, 5501 Hopkins | Review Boards, Reed Hall B- |
|  |  |  | Bayview Circle, Baltimore, | 130, 1620 McElderry Street, |
|  |  |  | Maryland, 21224, United States | Baltimore, Maryland, 21224, |
|  |  |  |  | Unites States |
|  |  |  |  | Chairperson: Carrese, Joseph |
|  |  |  |  |  |

| **Investigator** | **Sub-Investigator** | **Investigator** | **Description of Research** | **Name of IEC/IRB Committee,** |
| --- | --- | --- | --- | --- |
|  |  | **no./Center no.** | **Facility, Hospital/ Institution,** | **Address, Committee Chair** |
|  |  |  | **and Address** |  |
|  |  |  |  |  |
| Lorch Jr., Daniel G. | Cotten, Stephanie. RN | 016763/073981 | Pulmonary Associates of | Quorum Review, Inc., Suite |
| MD, CPI | Graves, Arthur E. MD |  | Brandon Clinical Research, Suite | 1000, 1601 Fifth Avenue, |
|  | Hooker, Thomas P. DO |  | 201, 910 Oakfield Drive, | Seattle, Washington, 98101, |
|  | Laneve, Theresa A. RN, BSN, |  | Brandon, Florida, 33511, United | United States. |
|  | CCRC |  | States |  |
|  | Poli, Francoise M. MA, CCRC |  |  | Chairperson: Kelley, David |
|  | Powell, Richard S. MD |  |  |  |
|  | Redd, L Danielle. MA |  |  |  |
|  | Shah, Suketu K. MD |  |  |  |
|  | Thompson, Andrea. LPN |  |  |  |
|  | Walton, Marcene A. MA |  |  |  |
|  |  |  |  |  |
| Mathur, Rameshwar N. | Mathur, Uma R. MD | 008405/073984 | Rameshwar Mathur, MD, PA, | Quorum Review, Inc., Suite |
| MD |  |  | 6250 North US Highway 1, | 1000, 1601 Fifth Avenue, |
|  |  |  | Cocoa, Florida, 32927, United | Seattle, Washington, 98101, |
|  |  |  | States | United States. |
|  |  |  |  | Chairperson: Kelley, David |
|  |  |  |  |  |

| **Investigator** | **Sub-Investigator** | **Investigator** | **Description of Research** | **Name of IEC/IRB Committee,** |
| --- | --- | --- | --- | --- |
|  |  | **no./Center no.** | **Facility, Hospital/ Institution,** | **Address, Committee Chair** |
|  |  |  | **and Address** |  |
|  |  |  |  |  |
| Mazdisnian, Farhad. | Sassoon, Catherine S. MD | 176326/073983 | VA Long Beach Health Care | VA Long Beach Health Care |
| MD |  |  | System, 11-111P, 5901 East 7th | System, 11-111P, 5901 East 7th |
|  |  |  | Street, Long Beach, California, | Street, Long Beach, 90822, |
|  |  |  | 90822, United States | California, United States |
|  |  |  |  | Chairperson: Herron, Kathleen |
|  |  |  |  |  |
| Moran, Joseph E. MD* | Hatharasinge, Roger A. MD | 030068/073986 | Carolina Pharmaceutical | Quorum Review, Inc., Suite |
|  | Hicks, Harlan M. MD |  | Research, 138 Sherlock Drive, | 1000, 1601 Fifth Avenue, |
|  | Stout III, Elmer H. MD |  | Statesville, North Carolina, | Seattle, Washington, 98101, |
|  |  |  | 28625, United States | United States |
|  |  |  |  | Chairperson: Kelley, David |
|  |  |  |  |  |
| Morganroth, Melvin. MD | Bowerfind, William M. MD | 019205/074081 | The Oregon Clinic, Suite 200, | Providence Health & Services |
|  | Chesnutt, Asha N. MD |  | 1111 NE 99th, Portland, Oregon, | Institutional Review Board, 525' |
|  | Cummings, Rhett J. MD |  | 97220, United States | NE Glisan, Bldg. A, 3rd Floor, |
|  | Hotchkin, David L. MD |  |  | Portland, Oregon, 97213, United |
|  | Jacobs, Marc A. MD |  |  | States |
|  |  |  |  |  |

| **Investigator** | **Sub-Investigator** | **Investigator** | **Description of Research** | **Name of IEC/IRB Committee,** |
| --- | --- | --- | --- | --- |
|  |  | **no./Center no.** | **Facility, Hospital/ Institution,** | **Address, Committee Chair** |
|  |  |  | **and Address** |  |
|  |  |  |  |  |
|  | Keppel, John F. MD |  |  |  |
|  | Lefor, Michael L. MD |  |  | Chairperson: Friedman, Eric |
|  | Libby, Louis S. MD |  |  |  |
|  | Schaumberg, Thomas H. MD |  |  |  |
|  | Schoen, Elke. FNP |  |  |  |
|  | Skokan, Michael D. MD |  |  |  |
|  | Strauss, Wayne L. MD |  |  |  |
|  | Taylor, Phyllis. FNP |  |  |  |
|  | Wesenberg, Karen J. MD |  |  |  |
|  |  |  |  |  |
| Morris, Felix A. MD | Mitchell, Jr., Ira L. MD | 100898/073987 | Florence Research Associates, | Quorum Review, Inc., Suite |
|  |  |  | LLC, Suite 2500, 416 North | 1000, 1601 Fifth Avenue, |
|  |  |  | Seminary Street, Florence, | Seattle, Washington, 98101, |
|  |  |  | Alabama, 35630, United States | United States |
|  |  |  |  | Chairperson: Kelley, David |
|  |  |  |  |  |

| **Investigator** | **Sub-Investigator** | **Investigator** | **Description of Research** | **Name of IEC/IRB Committee,** |
| --- | --- | --- | --- | --- |
|  |  | **no./Center no.** | **Facility, Hospital/ Institution,** | **Address, Committee Chair** |
|  |  |  | **and Address** |  |
|  |  |  |  |  |
| Nyanjom, David. MD | DeLeon, Fernando. MD | 177045/073990 | Pulmonary Disease & Critical | Quorum Review, Inc., Suite |
|  |  |  | Care Associates, PA, Suite 310, | 1000, 1601 Fifth Avenue, |
|  |  |  | 10710 Charter Drive, Columbia, | Seattle, Washington, 98101, |
|  |  |  | Maryland, 21044, United States | United States |
|  |  |  |  | Chairperson: Kelley, David |
|  |  |  |  |  |
| Ohar, Jill A. MD | Albon, Dana | 017098/073991 | Wake Forest Baptist Medical | Wake Forest University Health |
|  | Bellinger, Christina |  | Centre, 2132 Cloverdale | Sciences, Medical Center Blv, |
|  | Bleecker, Eugene |  | Avenue, Winston-Salem, North | 2132 Cloverdale Avenue, |
|  | Coleman, Jacob |  | Carolina, 27103, United States | Winston-Salem, 27157, North |
|  | Hatzis, Chris |  |  | Carolina, United States |
|  | Kahn, Irtaza |  |  |  |
|  | Moore, Wendy |  |  | Chairperson: Bertoni, Alain |
|  | Ortega, Victor |  |  |  |
|  | Pascual, Rudy |  |  |  |
|  | Peters, Stephen |  |  |  |
|  | Updaw, Robert |  |  |  |
|  |  |  |  |  |

| **Investigator** | **Sub-Investigator** | **Investigator** | **Description of Research** | **Name of IEC/IRB Committee,** |  |
| --- | --- | --- | --- | --- | --- |
|  |  | **no./Center no.** | **Facility, Hospital/ Institution,** | **Address, Committee Chair** |  |
|  |  |  | **and Address** |  |  |
|  |  |  |  |  |  |
| Panos, Ralph. MD | Eschenbacher, William. MD | 019782/073999 | Cincinnati VA Medical Center, | University of Cincinnati |  |
|  |  |  | 3200 Vine Street, Cincinnati, | Institutional Review Board, |  |
|  |  |  | Ohio, 45220, United States | University Hall, Suite 300, |  |
|  |  |  |  | 51 Goodman Drive, Cincinnati, |  |
|  |  |  |  | Ohio, 45221-0567, United States |  |
|  |  |  |  | Chairperson: Linke, Michael |  |
| Patel, Amit I. MD | Branch, John D. DO | 012037/074002 | Integrated Research Group, | Quorum Review, Inc., Suite |  |
|  | Canavan, Frank P. PA-C |  | Suite 202-3, 4646 Brockton | 1000, 1601 Fifth Avenue, |  |
|  | Davis III, Thomas L. MD |  | Avenue, Riverside, California, | Seattle, Washington, 98101, |  |
|  | Knopke III, Carl G. MD |  | 92506, United States | United States |  |
|  | Mikhail, Mina. MD |  |  | Chairperson: Kelley, David |  |
|  | Sindher, Raj K. MD |  |  |  |  |
|  |  |  |  |  |  |
|  |  |  |  |  |  |
| Pineda, Lilibeth. MD* | Robbins, Richard. MD | 074167/074084 | Phoenix VA Medical Center, | Phoenix VA Medical Center, |  |
|  |  |  | 650 East Indian School Road, | 111P, 650 East Indian School |  |
|  |  |  | Phoenix, Arizona, 85012, United | Road, Phoenix, Arizona, 85012, |  |
|  |  |  | States | United States |  |
|  |  |  |  | Chairperson: Mobley, Theodore |  |
|  |  |  |  |  |  |

| **Investigator** | **Sub-Investigator** | **Investigator** | **Description of Research** | **Name of IEC/IRB Committee,** |
| --- | --- | --- | --- | --- |
|  |  | **no./Center no.** | **Facility, Hospital/ Institution,** | **Address, Committee Chair** |
|  |  |  | **and Address** |  |
|  |  |  |  |  |
| Plautz, Mark. MD | Nauser, Trenton. MD | 130815/074004 | Veterans Affairs Medical Center | Kansas City Veterans Affairs |
|  | Perkins, Cheryl. APRN |  | #151, 4801 Linwood boulevard., | Medical Center IRB, Veterans |
|  |  |  | Kansas City, Missouri, 64128, | Affairs Medical Center #151, |
|  |  |  | United States | 4801 E Linwood boulevard, |
|  |  |  |  | Kansas City, Missouri, 64128, |
|  |  |  |  | United States |
|  |  |  |  | Chairperson: Sheedy, Carmen |
| Pudi, Krishna K. MD | Mansour, Abbas. MD | 009410/074006 | Upstate Pharmaceutical | Quorum Review, Inc., Suite |
|  | Williams, Julian. MD |  | Research, Suite C, 109 | 1000, 1601 Fifth Avenue, |
|  |  |  | Fleetwood Drive, Easley, South | Seattle, Washington, 98101, |
|  |  |  | Carolina, 29640, United States | United States |
|  |  |  |  | Chairperson: Kelley, David |
| Rastogi, Padmashri. | None | 136330/074015 | VA North Texas Health Care | VA North Texas Health Care |
| MD |  |  | System, 4500 South Lancaster, | System IRB (151), 4500 South |
|  |  |  | Dallas, Texas, 75216, United | Lancaster Road, Dallas, 75216, |
|  |  |  | States | Texas, United States |
|  |  |  |  | Chairperson: Dowell, Jonathan |

| **Investigator** | **Sub-Investigator** | **Investigator** | **Description of Research** | **Name of IEC/IRB Committee,** |  |
| --- | --- | --- | --- | --- | --- |
|  |  | **no./Center no.** | **Facility, Hospital/ Institution,** | **Address, Committee Chair** |  |
|  |  |  | **and Address** |  |  |
|  |  |  |  |  |  |
| Rennard, Stephen I. | Carlson, Mary. ARNP | 009021/075778 | University of Nebraska, | University of Nebraska Medical |  |
| MD* | Romberger, Debra J. MD |  | Pulmonary Clinical Studies Unit, | Center, 985456 Nebraska |  |
|  | Sitorius, Michael A. MD |  | 982465 Nebraska Medical | Medical Center, Omaha, |  |
|  | Tape, Thomas G. MD |  | Center, Omaha, Nebraska, | Nebraska, 68198, United States |  |
|  | Thierfelder, Jean L. MD |  | 68198, United States | Chairperson: Prentice, Ernest |  |
|  | Vasquez, Eduardo. MD |  |  |  |  |
|  |  |  |  |  |  |
|  |  |  |  |  |  |
| Richmond, Gary J. MD | Appleby, Vernon F. RN | 014588/074018 | Gary J. Richmond, MD , PA, 315 | Quorum Review, Inc., Suite |  |
|  |  |  | SE 14th Street, Fort Lauderdale, | 1000, 1601 Fifth Avenue, |  |
|  |  |  | Florida, 33316, United States | Seattle, Washington, 98101, |  |
|  |  |  |  | United States |  |
|  |  |  |  | Chairperson: Kelley, David |  |
|  |  |  |  |  |  |
| Robinette, Jr., Emory H. | Hawkins, Andrew F. MD | 009507/074019 | Pulmonary Research of | Quorum Review, Inc., Suite |  |
| MD |  |  | Abingdon, 223 East Valley | 1000, 1601 Fifth Avenue, |  |
|  |  |  | Street, Abingdon, Virginia, | Seattle, Washington, 98101, |  |
|  |  |  | 24210, United States | United States |  |
|  |  |  |  | Chairperson: Kelley, David |  |
|  |  |  |  |  |  |

| **Investigator** | **Sub-Investigator** | **Investigator** | **Description of Research** | **Name of IEC/IRB Committee,** |
| --- | --- | --- | --- | --- |
|  |  | **no./Center no.** | **Facility, Hospital/ Institution,** | **Address, Committee Chair** |
|  |  |  | **and Address** |  |
|  |  |  |  |  |
| Rojas-Caballero, Julian | Lucht, William D. MD | 211646/080372 | Lung & Sleep Clinic of Alaska, | Quorum Review, Inc., Suite |
| T. MD |  |  | Inc., Suite C-307, 2741 De Barr | 1000, 1601 Fifth Avenue, |
|  |  |  | Road, Anchorage, Alaska, | Seattle, Washington, 98101, |
|  |  |  | 99508, United States | United States |
|  |  |  |  | Chairperson: Kelley, David |
|  |  |  |  |  |
| Salzman, Gary A. MD | Bhat, Abid M. MD | 017208/074020 | Truman Medical Center, 2301 | University of Missouri - Kansas |
|  | Gohar, Ashraf. MD |  | Holmes Street, Kansas City, | City, 5319 Rockhill Road, |
|  | Ladesich, James B. MD |  | Missouri, 64108, United States | Kansas City, Missouri, 64110, |
|  | Pyszczynski, Dennis R. MD |  |  | United States |
|  |  |  |  | Chairperson: Sommi, Roger |
|  |  |  |  |  |
| Seibert, Allan F. MD | Bedsole, Donald Lawrence. | 006948/074038 | Pulmonary Associates of Mobile | Quorum Review, Inc., Suite |
|  | MD |  | PC, Suite B131, 6701 Airport | 1000, 1601 Fifth Avenue, |
|  | Bekurs, Sada C. RN |  | boulevard, Mobile, Alabama, | Seattle, Washington, 98101, |
|  | Cowan, Greg A. RRT, CCRC |  | 36608, United States | United States |
|  | Craig, Letoya P |  |  |  |
|  |  |  |  |  |

| **Investigator** | **Sub-Investigator** | **Investigator** | **Description of Research** | **Name of IEC/IRB Committee,** |
| --- | --- | --- | --- | --- |
|  |  | **no./Center no.** | **Facility, Hospital/ Institution,** | **Address, Committee Chair** |
|  |  |  | **and Address** |  |
|  |  |  |  |  |
|  | Hemphill, Michele L. RN, |  |  | Chairperson: Kelley, David |
|  | CCRC |  |  |  |
|  | Huffmaster, Beth. RN, BSN |  |  |  |
|  | Melton, Casey W. MD |  |  |  |
|  | McLeod Saizan, Jennifer N. |  |  |  |
|  | RN, BSN |  |  |  |
|  | Sindel, Lawrence J. MD |  |  |  |
|  |  |  |  |  |
| Sethi, Sanjay. MD, | Provost, Karin A. DO, PhD, | 009877/075282 | VA Western NY Healthcare | VA Western NY Healthcare |
| FACP* | FCCP |  | System, 3495 Bailey Avenue, | System, 3495 Bailey Avenue, |
|  |  |  | Buffalo, New York, 14215-1199, | Buffalo, New York, 14215-1199, |
|  |  |  | United States | United States |
|  |  |  |  | Chairperson: Wray, Laura |
| Shah, Amit G. MD | Patel, Nimish K. MD | 219438/083882 | Piedmont Research Partners, | Quorum Review, Inc., Suite |
|  | Sprinkle, Brenda B. PA-C |  | Suite 1600, 9789 Charlotte Hwy., | 1000, 1601 Fifth Avenue, |
|  |  |  | Fort Mill, South Carolina, 29707, | Seattle, Washington, 98101, |
|  |  |  | United States | United States |
|  |  |  |  | Chairperson: Kelley, David |
|  |  |  |  |  |

| **Investigator** | **Sub-Investigator** | **Investigator** | **Description of Research** | **Name of IEC/IRB Committee,** |
| --- | --- | --- | --- | --- |
|  |  | **no./Center no.** | **Facility, Hospital/ Institution,** | **Address, Committee Chair** |
|  |  |  | **and Address** |  |
|  |  |  |  |  |
| Sharafkhaneh, Amir. | Lan, Charlie. DO, DABSM | 021093/074039 | Veterans Affairs Medical Center, | Baylor College of Medicine, |
| MD, PhD* |  |  | Houston, VAMC - 111, 2002 | Institutional Review Board, One |
|  |  |  | Holcombe Boulevard, Houston, | Baylor Plaza, Room 600D, |
|  |  |  | Texas, 77030, United States | Houston, Texas, 77030, United |
|  |  |  |  | States |
|  |  |  |  | Chairperson: Habib, Gabriel |
|  |  |  |  |  |
| Shofer, Scott L. MD, | Wahidi, Momen H. MD, MBA | 196857/074113 | Duke University Hospital, 2301 | Duke University Health System |
| PhD |  |  | Erwin Rd., Durham, North | IRB, Ste. 405, 2424 Erwin Road, |
|  |  |  | Carolina, 27705, United States | Durham, North Carolina, 27710, |
|  |  |  |  | United States |
|  |  |  |  | Chairperson: Falleta, John |
|  |  |  |  |  |
| Sigal, Barry W. MD, | Bonnett, Kristie D. RN-MSN, | 009125/074375 | Southeastern Research Center, | Quorum Review, Inc., Suite |
| FCCP | ARPNP-BC, CCRC |  | LLC, 3019 Lyndhurst Avenue, | 1000, 1601 Fifth Avenue, |
|  | Gallup Jr., Kenneth R. MD |  | Winston-Salem, North Carolina, | Seattle, Washington, 98101, |
|  | Pearsall, Luke A. PA-C |  | 27103, United States | United States |
|  |  |  |  |  |

| **Investigator** | **Sub-Investigator** | **Investigator** | **Description of Research** | **Name of IEC/IRB Committee,** |
| --- | --- | --- | --- | --- |
|  |  | **no./Center no.** | **Facility, Hospital/ Institution,** | **Address, Committee Chair** |
|  |  |  | **and Address** |  |
|  |  |  |  |  |
|  | Smith, Margaret H. PA-C |  |  |  |
|  | Thomason, Jason W. MD, |  |  | Chairperson: Kelley, David |
|  | FCCP, D-ABSM |  |  |  |
|  | Wilmoth, Jennifer E. PA-C |  |  |  |
|  |  |  |  |  |
| Sinkowitz, Deren. MD | Destasio, Shawn. FNP-C | 077984/074043 | Peninsula Pulmonary Medical | Quorum Review, Inc., Suite |
|  | Eltawil, Khalid. MD |  | Associates, Suite 180, 23550 | 1000, 1601 Fifth Avenue, |
|  | Mimura, Keiko. GNP-C |  | Hawthorne Blvd, Torrance, | Seattle, Washington, 98101, |
|  |  |  | California, 90505, United States | United States |
|  |  |  |  | Chairperson: Kelley, David |
|  |  |  |  |  |
| Southard, John G. MD, | Rogers, Kathie Z. PhD | 196947/074114 | Loess Hill Research Center, | Quorum Review, Inc., Suite |
| PhD |  |  | #501, 933 E Pierce ST, Council | 1000, 1601 Fifth Avenue, |
|  |  |  | Bluffs, Iowa, 51503, United | Seattle, Washington, 98101, |
|  |  |  | States | United States |
|  |  |  |  | Chairperson: Kelley, David |
|  |  |  |  |  |

| **Investigator** | **Sub-Investigator** | **Investigator** | **Description of Research** | **Name of IEC/IRB Committee,** |
| --- | --- | --- | --- | --- |
|  |  | **no./Center no.** | **Facility, Hospital/ Institution,** | **Address, Committee Chair** |
|  |  |  | **and Address** |  |
|  |  |  |  |  |
| Spangenthal, Selwyn. | Dinger, Shawna. PA-C | 018106/074044 | American Health Research, | Quorum Review, Inc., Suite |
| MD | Giordano, Joseph A. RRT, PA- |  | 1918 Randolph Road Suite 440, | 1000, 1601 Fifth Avenue, |
|  | C |  | Charlotte, North Carolina, 28207, | Seattle, Washington, 98101, |
|  | Glenn, Stephanie M. MD |  | United States | United States |
|  | Schmidlin, Christine. PA-C |  |  |  |
|  | Tam, Kim H. MD |  |  | Chairperson: Kelley, David |
|  |  |  |  |  |
| Specht, Norman L. MD | Ellstrom, Kathleen. PhD, RN, | 197009/074233 | VA Loma Linda Healthcare | VA Loma Linda Healthcare |
|  | ACNS-BC |  | System, 11201 Benton Street, | System, Institutional Review |
|  |  |  | Loma Linda, California 92357, | Board # I - (151), 11201 Benton |
|  |  |  | United States | Street, Lorna Linda, California |
|  |  |  |  | 92357, Unites States |
|  |  |  |  | Chairperson: Jennings, John |
|  |  |  |  |  |
| Sriram, Peruvemba S. | Berry, Richard. MD | 176676/082740 | North Florida/South Georgia | University of Florida Institutional |
| MD | Wynne, James. MD |  | Veterans Health System, | Review Board – 01, Gainesville |
|  |  |  | Malcom Randall VAMC, 1601 | Health Science Center, P.O. Box |
|  |  |  | SW Archer (151), Gainesville, | 100173, 1600 SW Archer Road, |
|  |  |  |  |  |

| **Investigator** | **Sub-Investigator** | **Investigator** | **Description of Research** | **Name of IEC/IRB Committee,** |
| --- | --- | --- | --- | --- |
|  |  | **no./Center no.** | **Facility, Hospital/ Institution,** | **Address, Committee Chair** |
|  |  |  | **and Address** |  |
|  |  |  |  |  |
|  |  |  | Florida, 32608m, United States | Gainesville, Florida, 32610-0173 |
|  |  |  |  | United States |
|  |  |  |  | Chairperson: Iafrate, Peter |
|  |  |  |  |  |
| Trevino, Miguel. MD | Burke, Deborah. MD | 064621/074048 | Innovative Research of West | Quorum Review, Inc., Suite |
|  | Segarra, Robert. PA-C |  | Florida, Inc., 1573 South Fort | 1000, 1601 Fifth Avenue, |
|  |  |  | Harrison Ave, Clearwater, | Seattle, Washington, 98101, |
|  |  |  | Florida, 33756, United States | United States |
|  |  |  |  | Chairperson: Kelley, David |
|  |  |  |  |  |
| Updegrove, John D. | Driver Merida, Janquilyn. FNP | 022391/074049 | Corsicana Medical Research, | Quorum Review, Inc., Suite |
| MD |  |  | PLLC, Suite #165, 301 Hospital | 1000, 1601 Fifth Avenue, |
|  |  |  | Drive, Corsicana, Texas, 75110, | Seattle, Washington, 98101, |
|  |  |  | United States | United States |
|  |  |  |  | Chairperson: Kelley, David |
|  |  |  |  |  |

| **Investigator** | **Sub-Investigator** | **Investigator** | **Description of Research** | **Name of IEC/IRB Committee,** |
| --- | --- | --- | --- | --- |
|  |  | **no./Center no.** | **Facility, Hospital/ Institution,** | **Address, Committee Chair** |
|  |  |  | **and Address** |  |
|  |  |  |  |  |
| Urdaneta-Jaimes, Jose | Ameh, Joseph. MD | 177047/074050 | Pulmonary Disease Research, | Quorum Review, Inc., Suite |
| A. MD, FCCP, FAASM | Daniels, Heather |  | Suite B, 1121 North Central | 1000, 1601 Fifth Avenue, |
|  | Griggs, Adam L. DO, FCCP |  | Avenue, Kissimmee, Florida, | Seattle, Washington, 98101, |
|  | Lucio, James A. MD, FCCP |  | 34741, United States | United States |
|  | Metcalf, Susan A. ARNP-BC |  |  |  |
|  | Nazario, Roderick C. MD, |  |  | Chairperson: Kelley, David |
|  | FCCP |  |  |  |
|  | Theriault, Vivian M. ARNP-BC, |  |  |  |
|  | MS, CCRN |  |  |  |
|  |  |  |  |  |
| Waldman, Lawrence F. | Mollen, Martin D. MD | 160896/086425 | Arizona Research Center, LLC, | Quorum Review, Inc., Suite |
| MD* |  |  | Suite 114, 2525 W. Greenway | 1000, 1601 Fifth Avenue, |
|  |  |  | Road, Phoenix, Arizona, 85023, | Seattle, Washington, 98101, |
|  |  |  | United States | United States |
|  |  |  |  | Chairperson: Kelley, David |
|  |  |  |  |  |

| **Investigator** | **Sub-Investigator** | **Investigator** | **Description of Research** | **Name of IEC/IRB Committee,** |
| --- | --- | --- | --- | --- |
|  |  | **no./Center no.** | **Facility, Hospital/ Institution,** | **Address, Committee Chair** |
|  |  |  | **and Address** |  |
|  |  |  |  |  |
| Weinberg, Paul B. MD | Brown, Chevy J. MPH, CCRC | 017249/074822 | Gwinnett Biomedical Research, | Quorum Review, Inc., Suite |
|  | Brown Jr., George A. BS |  | Suite 160, 600 Professional | 1000, 1601 Fifth Avenue, |
|  | Feldman, David. BBA, CCRC |  | Drive, Lawrenceville, Georgia, | Seattle, Washington, 98101, |
|  | Fisher, Amy |  | 30046, United States | United States |
|  | Garimella, Prasad S. MD |  |  |  |
|  | Gross, Suvi. BA |  |  | Chairperson: Kelley, David |
|  | Hain, Aldin. BSBA, CNA |  |  |  |
|  | Jasani, Rajesh R. MD |  |  |  |
|  | Kaplan, Larry (Lawrence) D. |  |  |  |
|  | MD |  |  |  |
|  | Lozynsky, Regina. LPN |  |  |  |
|  | McGann, Jr., William F. MD |  |  |  |
|  | Nisbet, Rachel E. MD |  |  |  |
|  | Sineway, Michael. MD |  |  |  |
|  | Weinberg, Amanda. BA |  |  |  |
|  |  |  |  |  |

| **Investigator** | **Sub-Investigator** | **Investigator** | **Description of Research** | **Name of IEC/IRB Committee,** |  |
| --- | --- | --- | --- | --- | --- |
|  |  | **no./Center no.** | **Facility, Hospital/ Institution,** | **Address, Committee Chair** |  |
|  |  |  | **and Address** |  |  |
|  |  |  |  |  |  |
| Weinstein, Gary L. MD* | Rousseau, Wyatt E. MD | 051571/074698 | Texas Health Research & | Texas Health Resources IRB, |  |
|  |  |  | Education Institute, Suite 518, | Ste. 1212, 612 E. Lamar |  |
|  |  |  | 8230 Walnut Hill Lane, Dallas, | Boulevard, Arlington, Texas, |  |
|  |  |  | Texas, 75231, United States | 76011, United States |  |
|  |  |  |  | Chairperson: Berk, Martin |  |
| Yunger Jr., Thomas M. | Goyal, Anuj. MD, FCCP, | 177332/074054 | Midwest Pulmonary and sleep | Quorum Review, Inc., Suite |  |
| MD, FCCP, DABSM | DABSM |  | Research, 7056 Corporate Way, | 1000, 1601 Fifth Avenue, |  |
|  | Mathews, Judy E. RN |  | Dayton, Ohio, 45459, United | Seattle, Washington, 98101, |  |
|  | Patel, Rajesh C. MD, FCCP, |  | States | United States |  |
|  | DABSM |  |  | Chairperson: Kelley, David |  |
|  |  |  |  |  |  |
| Zeidler, Michelle R. MD | Leung, Felix W. MD | 018605/073979 | VA Greater los Angeles | VA Greater Los Angeles |  |
|  | Littner, Michael. MD |  | HealthCare System, 11301 | Healthcare System, |  |
|  | Santiago, Silverio. MD |  | Wilshire Boulevard (111 Q), Los | 11301 Wilshire Boulevard, Los |  |
|  |  |  | Angeles, California, 90073, | Angeles, California, 90073, |  |
|  |  |  | United states | United States |  |
|  |  |  |  | Chairperson: Shekelle, Paul |  |

| **Investigator** | **Sub-Investigator** | **Investigator** | **Description of Research** | **Name of IEC/IRB Committee,** |
| --- | --- | --- | --- | --- |
|  |  | **no./Center no.** | **Facility, Hospital/ Institution,** | **Address, Committee Chair** |
|  |  |  | **and Address** |  |
|  |  |  |  |  |
| ZuWallack, Richard. | Gerardi, Daniel. MD | 012252/074055 | Saint Francis Hospital and | Saint Francis Hospital and |
| MD* | Grover, Prashant. MD |  | Medical Center, 114 Woodland | Medical Center, 114 Woodland |
|  |  |  | Street, Hartford, Connecticut, | Street, Hartford, Connecticut, |
|  |  |  | 6105, United States | 06105, United States |
|  |  |  |  | Chairperson: Canalis, Ernesto |
|  |  |  |  |  |
| *No subjects enrolled. |  |  |  |  |
| All centers participated in the study under the US IND. | |  |  |  |
