# Supplementary material for: Fluticasone propionate/salmeterol 250/50 μg versus salmeterol 50 μg after chronic obstructive pulmonary disease exacerbation
Source: Respir Res. 2014 Sep 24;15(1):105. doi: 10.1186/s12931-014-0105-2 (PMC4176847; doi:10.1186/s12931-014-0105-2)
Supplement: Additional file 3: — Statistical methods used in the analysis of other efficacy endpoints. [file 12931_2014_105_MOESM3_ESM.docx]

**Fluticasone Propionate/Salmeterol 250/50µg Versus Salmeterol 50µg After Chronic Obstructive Pulmonary Disease Exacerbation**

**Authors:** *Jill A. Ohar, MD; Glenn D. Crater, MD; Amanda Emmett, MS; Thomas J. Ferro, MD; Andrea N. Morris, BSN; Ibrahim Raphiou, PhD; P.S. Sriram, MD; and Mark T. Dransfield, MD*

Supplemental Material

# Additional file 3: Statistical methods used in the analysis of other efficacy endpoints

**Biomarkers SP-D, CC-16 and hs-CRP:** Surfactant protein D (SP-D) was included as a biomarker of pulmonary injury.^1^ Clara cell secretory protein 16 (CC-16) was included as a potential surrogate marker of non-ciliate epithelial dysfunction.^2^ High-sensitivity reactive protein (hs-CRP) was included as an indicator of systemic inflammation.^3^ The primary analysis of each biomarker was mean change from baseline compared between treatment groups at Endpoint. As SP-D, CC-16 and hs-CRP were non-normally distributed, the biomarker values were log-transformed prior to analysis. Endpoint was defined as the last measurement during the 26-week treatment period after the 21-day stabilization period, and baseline was defined as the biomarker measure from Randomization (Visit 2). Log-transformed SP-D, CC-16, hs-CRP values and change from baseline values were summarized with descriptive statistics by treatment group and an analysis of covariance (ANCOVA) model including terms for treatment group, pooled investigator, randomization stratum and baseline was used to test statistical differences between treatment groups at Endpoint and each visit (Visits 3, 4 and 5, Weeks 0, 13 and 26, respectively).

**Time to first chronic obstructive pulmonary disease (COPD) exacerbation requiring treatment with oral corticosteroids (OCS), antibiotics and/or hospitalization:**  Time to first COPD exacerbation requiring treatment with OCS, antibiotics and/or hospitalization was summarized by treatment group using Kaplan-Meier estimates. A Cox proportional hazards regression model was used to compare time to first exacerbation between treatment groups. The hazard ratio for the treatment comparison was derived from a Cox proportional hazards model with terms for baseline disease severity (baseline forced expiratory volume in 1 second [FEV_1_] percent predicted), pooled investigator, randomization stratum and treatment group in the model.

**Pre-dose FEV_1_:** The primary analysis of pre-dose FEV_1_ was mean change from baseline compared between treatment groups at Endpoint. Endpoint was defined as the last scheduled measurement of pre-dose FEV_1_ during the 26-week treatment period after the 21-day stabilization period, and baseline was defined as the pre-dose FEV_1_ measure from Randomization (Visit 2). FEV_1_ measures and change from baseline values were summarized with descriptive statistics by treatment group and an ANCOVA model including terms for treatment group, pooled investigator, randomization stratum and baseline was used to test statistical differences between treatment groups at Endpoint and each visit (Visits 3 and 5, Weeks 0 and 26, respectively).

**Probability of study withdrawal:** The probability of study withdrawal was summarized with Kaplan-Meier estimates and compared between treatment groups using a Cox proportional hazards regression model with terms for baseline severity (baseline FEV_1_ percent predicted), pooled investigator, randomization stratum and treatment group in the model.

**Health Outcomes Analysis:**

***Chronic respiratory questionnaire - self-administered standardized (CRQ-SAS):*** The CRQ-SAS was administered at Randomization (Visit 2) and at all subsequent visits including the Early Withdrawal visit. Baseline was determined from the Randomization (Visit 2) questionnaire and Endpoint was determined from the last questionnaire collected during the treatment period or at the Early Withdrawal visit. The primary analysis was mean change from baseline scores compared between treatment groups at Endpoint for each of four CRQ-SAS domains (dyspnea, fatigue, emotional function, and mastery). CRQ-SAS domain scores and change from baseline values were summarized with descriptive statistics at Endpoint and each visit for which the CRQ-SAS was administered. An ANCOVA model, including terms for treatment group, pooled investigator, randomization stratum and baseline severity (baseline FEV_1_ percent predicted), was used to test statistical differences in treatment group mean changes from baseline at Endpoint and each visit.

***Exacerbations of chronic pulmonary disease tool – patient reported outcomes (EXACT-PRO):*** The EXACT-PRO Total score and three respiratory symptom domain scores (breathlessness, cough and sputum, and chest symptoms) were each scored on a 0–100 scale with higher scores indicating a more severe COPD state. The primary analysis compared mean total and domain scores between treatment groups at Endpoint. Mean total and domain scores were summarized with descriptive statistics by treatment group at Endpoint, for the overall 26-week treatment period after the 3-week stabilization period, and for each of the following time periods: Weeks 1–4, 5–8, 9–12, 13–16, 17–20, 21–24, and 25–26. An ANCOVA model, including terms for treatment group, pooled investigator, randomization stratum and baseline severity (baseline FEV_1_ percent predicted), was used to test statistical differences in treatment group means at Endpoint, for the overall treatment period, and each 4-week time period.

**References for e-Appendix 3:**

1. Bowler RP. Surfactant Protein D as a biomarker for chronic obstructive pulmonary disease. *COPD.* 2012;9(6):651-653.
2. Dickens JA, Lomas DA. CC-16 as a biomarker in chronic obstructive pulmonary disease. *COPD.* 2012;9(5):574-575.
3. Piehl-Aulin K, Jones I, Lindvall B, Magnuson A, Abdel-Halim SM. Increased serum inflammatory markers in the absence of clinical and skeletal muscle inflammation in patients with chronic obstructive pulmonary disease. *Respiration.* 2009;78(2):191-196.
