# Supplementary material for: Fluticasone propionate/salmeterol 250/50 μg versus salmeterol 50 μg after chronic obstructive pulmonary disease exacerbation
Source: Respir Res. 2014 Sep 24;15(1):105. doi: 10.1186/s12931-014-0105-2 (PMC4176847; doi:10.1186/s12931-014-0105-2)
Supplement: Additional file 4: Table S1. — CRQ-SAS Change from Baseline at 3 + 26-Week Study Endpoint and EXACT-PRO Total Score and Components at Study Endpoint, ITT Population. [file 12931_2014_105_MOESM4_ESM.docx]

**Fluticasone Propionate/Salmeterol 250/50µg Versus Salmeterol 50µg After Chronic Obstructive Pulmonary Disease Exacerbation**

**Authors:** *Jill A. Ohar, MD; Glenn D. Crater, MD; Amanda Emmett, MS; Thomas J. Ferro, MD; Andrea N. Morris, BSN; Ibrahim Raphiou, PhD; P.S. Sriram, MD; and Mark T. Dransfield, MD*

**Additional file 4: Table S1—*CRQ-SAS Change from Baseline at 3+26-Week Study Endpoint and EXACT-PRO Total Score and Components at Study Endpoint, ITT Population***

|  | | | | FP/SAL 250/50  (N=314) | SAL 50  (N=325) | LS Mean Diff. (SE) | 95% CI | |
| --- | --- | --- | --- | --- | --- | --- | --- | --- |
| CRQ-SAS (domains) | | | | | | | | |
| Change from baseline | Mastery | n  Mean (SE) | | 286  0.99 (0.094) | 291  0.85 (0.093) | 0.04  (0.113) | (–0.18, 0.26) | |
|  | Fatigue | n  Mean (SE) | | 286  0.90 (0.088) | 291  0.80 (0.076) | 0.09  (0.100) | (–0.11, 0.29) | |
|  | Emotional function | n  Mean (SE) | | 286  0.89 (0.083) | 291  0.77 (0.078) | 0.08  (0.096) | (–0.11, 0.27) | |
|  | Dyspnea | n  Mean (SE) | | 285  0.73 (0.095) | 291  0.74 (0.088) | 0.04  (0.111) | (–0.18, 0.25) | |
| EXACT-PRO (Total and components) | | | | | | | |  |
| Total score | Total | | n  Mean (SE) | 294  35.9 (0.91) | 302  36.4 (0.89) | –0.6  (1.17) | (–2.8, 1.7) |  |
|  | Breathless-ness | | n  Mean (SE) | 294  38.7 (1.20) | 302  40.3 (1.25) | –1.8  (1.56) | (–4.9, 1.2) |  |
|  | Cough and sputum | | n  Mean (SE) | 294  26.0 (1.09) | 302  26.9 (1.08) | –1.0  (1.47) | (–3.9, 1.9) |  |
|  | Chest symptoms | | n  Mean (SE) | 294  27.1 (1.22) | 302  26.2 (1.27) | 0.7  (1.67) | (–2.6, 4.0) |  |
| LS mean diff., SE and CI are from an ANCOVA model with terms for treatment, country, randomization stratum and baseline. LS mean differences are calculated as FP/SAL 250/50 - SAL 50.  ANCOVA = analysis of covariance; CI = confidence interval; CRQ-SAS = chronic respiratory questionnaire - self-administered standardized; EXACT-PRO = exacerbations of chronic pulmonary disease tool – patient reported outcomes; FP = fluticasone propionate; ITT = intent-to-treat; LS = least squares; SAL = salmeterol; SE = standard error | | | | | | | | |
