# Supplementary material for: Fluticasone propionate/salmeterol 250/50 μg versus salmeterol 50 μg after chronic obstructive pulmonary disease exacerbation
Source: Respir Res. 2014 Sep 24;15(1):105. doi: 10.1186/s12931-014-0105-2 (PMC4176847; doi:10.1186/s12931-014-0105-2)
Supplement: Additional file 5: Table S2. — Change from Baseline CRQ-SAS Domain Scores and EXACT-PRO Total Scores at Treatment Period Weeks 13 and 26 and at 26-Week Endpoint for Patients Having 0 or ≥1 On-Treatment Exacerbations, ITT Population. [file 12931_2014_105_MOESM5_ESM.docx]

**Fluticasone Propionate/Salmeterol 250/50µg Versus Salmeterol 50µg After Chronic Obstructive Pulmonary Disease Exacerbation**

**Authors:** *Jill A. Ohar, MD; Glenn D. Crater, MD; Amanda Emmett, MS; Thomas J. Ferro, MD; Andrea N. Morris, BSN; Ibrahim Raphiou, PhD; P.S. Sriram, MD; and Mark T. Dransfield, MD*

**Additional file 5: Table S2—*Change from Baseline CRQ-SAS Domain Scores and EXACT-PRO Total Scores at Treatment Period Weeks 13 and 26 and at 26-Week Endpoint for Patients Having 0 or ≥1 On-Treatment Exacerbations, ITT Population***

|  | | | ≥1  On-Treatment Exacerbations (N=217) | No  On-Treatment Exacerbation (N=422) | LS Mean Diff. (SE) | 95% CI |
| --- | --- | --- | --- | --- | --- | --- |
| Week 13 | | | | | | |
| Change from baseline | Mastery | n  Mean (SE) | 168  0.90 (0.116) | 250  0.99 (0.101) | –0.28  (0.134) | (–0.54, –0.02) |
|  | Fatigue | n  Mean (SE) | 168  0.80 (0.102) | 250  0.96 (0.089) | –0.31  (0.120) | (–0.54, –0.07) |
|  | Emotional function | n  Mean (SE) | 168  0.81 (0.102) | 250  0.91 (0.081) | –0.21  (0.113) | (–0.44, 0.01) |
|  | Dyspnea | n  Mean (SE) | 168  0.69 (0.125) | 249  0.99 (0.101) | –0.58  (0.132) | (–0.84, –0.32) |
| Total score | EXACT-PRO Total Score^a^ | n  mean (SE) | 189 38.7 (0.91) | 268 32.0 (0.87) | 5.4  (1.24) | (2.9, 7.8) |
| Week 26 | | | | | | |
| Change from baseline | Mastery | n  Mean (SE) | 145  1.11 (0.119) | 242  1.22 (0.108) | –0.35  (0.138) | (–0.63, –0.08) |
|  | Fatigue | n  Mean (SE) | 145  1.05 (0.100) | 242  1.03 (0.096) | –0.14  (0.125) | (–0.38, 0.11) |
|  | Emotional function | n  Mean (SE) | 145  1.02 (0.108) | 242  1.05 (0.088) | –0.18  (0.119) | (–0.41, 0.06) |
|  | Dyspnea | n  Mean (SE) | 145  0.75 (0.127) | 242  0.99 (0.100) | –0.53  (0.137) | (–0.80, –0.26) |
| Total score | EXACT-PRO Total Score^b^ | n  mean (SE) | 95 37.5 (1.48) | 137 30.5 (1.28) | 5.2  (1.90) | (1.5, 9.0) |
| Endpoint | | | | | | |
| Change from baseline | Mastery | n  Mean (SE) | 171  0.99 (0.109) | 258  1.18 (0.103) | –0.38  (0.129) | (–0.64, –0.13) |
|  | Fatigue | n  Mean (SE) | 171  0.96 (0.092) | 258  1.02 (0.091) | –0.19  (0.116) | (–0.42, 0.04) |
|  | Emotional function | n  Mean (SE) | 171  0.93 (0.100) | 258  1.04 (0.085) | –0.22  (0.111) | (–0.44, 0.00) |
|  | Dyspnea | n  Mean (SE) | 171  0.69 (0.123) | 257  0.97 (0.096) | –0.55  (0.129) | (–0.80, –0.29) |
| Total score | EXACT-PRO Total Score^c^ | n  mean (SE) | 213 39.4 (1.06) | 304 31.7 (0.89) | 6.0  (1.30) | (3.4, 8.5) |
| LS mean diff., SE and CI are from an ANCOVA model. LS mean differences are calculated as ≥1 exacerbation – 0 exacerbations.  ANCOVA = analysis of covariance; CI = confidence interval; CRQ-SAS = chronic respiratory questionnaire - self-administered standardized; EXACT-PRO = exacerbations of chronic pulmonary disease tool – patient reported outcomes; ITT = intent-to-treat; LS = least squares; SE = standard error.  ^a^Score for Weeks 9–12.  ^b^Score for Weeks 25–26.  ^c^Score for 26-week endpoint. | | | | | | |
