# Supplementary material for: Fluticasone propionate/salmeterol 250/50 μg versus salmeterol 50 μg after chronic obstructive pulmonary disease exacerbation
Source: Respir Res. 2014 Sep 24;15(1):105. doi: 10.1186/s12931-014-0105-2 (PMC4176847; doi:10.1186/s12931-014-0105-2)
Supplement: Additional file 6: Table S3. — Change from Baseline at 3 + 26-Week Study Endpoint for Biomarkers of Systemic Inflammation, ITT Population. [file 12931_2014_105_MOESM6_ESM.docx]

**Fluticasone Propionate/Salmeterol 250/50µg Versus Salmeterol 50µg After Chronic Obstructive Pulmonary Disease Exacerbation**

**Authors:** *Jill A. Ohar, MD; Glenn D. Crater, MD; Amanda Emmett, MS; Thomas J. Ferro, MD; Andrea N. Morris, BSN; Ibrahim Raphiou, PhD; P.S. Sriram, MD; and Mark T. Dransfield, MD*

**Additional file 6: Table S3—*Change from Baseline at 3+26-Week Study Endpoint for Biomarkers of Systemic Inflammation, ITT Population***

|  | | | FP/SAL 250/50  (N=314) | SAL 50  (N=325) | LS Mean Diff.  (SE) | 95% CI | |
| --- | --- | --- | --- | --- | --- | --- | --- |
| Summary analysis of hs-CRP | | | | | | | |
| Baseline | log hs-CRP | n  Mean (SE) | 303  1.41 (0.078) | 311  1.37 (0.083) |  |  | |
| Endpoint | log hs-CRP | n  Mean (SE) | 302  1.42 (0.072) | 299  1.46 (0.074) | –0.04  (0.099) | (–0.23, 0.16) | |
|  | Change from baseline | n  Mean (SE) | 292  0.04 (0.090) | 290  0.09 (0.096) |  |  |  |
| Summary analysis of SP-D | | | | | | |  |
| Baseline | log SP-D | n  Mean (SE) | 293  4.60 (0.044) | 309  4.64 (0.050) |  |  |  |
| Endpoint | log SP-D | n  Mean (SE) | 298  4.93 (0.040) | 300  5.00 (0.042) | –0.04  (0.047) | (–0.13, 0.05) |  |
|  | Change from baseline | n  Mean (SE) | 281  0.34 (0.039) | 288  0.37 (0.041) |  |  |  |
| Summary analysis of CC-16 | | | | | | |  |
| Baseline | log CC-16 | n  Mean (SE) | 310  1.66 (0.039) | 320  1.66 (0.038) |  |  |  |
| Endpoint | log CC-16 | n  Mean (SE) | 299  1.59 (0.042) | 302  1.58 (0.043) | <0.01  (0.040) | (–0.07, 0.08) |  |
|  | Change from baseline | n  Mean (SE) | 296  -0.08 (0.030) | 300  -0.08 (0.029) |  |  |  |
| LS mean diff., SE and CI are from an ANCOVA model with terms for treatment, country, randomization stratum and baseline value. LS mean differences are calculated as FP/SAL 250/50 – SAL 50.  ANCOVA = analysis of covariance; CI = confidence interval; CC-16 = Clara Cell secretory protein 16; FP = fluticasone propionate; hs-CRP = high-sensitivity C-reactive protein; ITT = intent-to-treat; LS = least squares; SAL = salmeterol; SE = standard error; SP-D = surfactant protein D. | | | | | | | |
