# Supplementary material for: Fluticasone propionate/salmeterol 250/50 μg versus salmeterol 50 μg after chronic obstructive pulmonary disease exacerbation
Source: Respir Res. 2014 Sep 24;15(1):105. doi: 10.1186/s12931-014-0105-2 (PMC4176847; doi:10.1186/s12931-014-0105-2)
Supplement: Additional file 7: — On- and post-treatment serious and fatal adverse events and incidences of pneumonia. [file 12931_2014_105_MOESM7_ESM.docx]

**Fluticasone Propionate/Salmeterol 250/50µg Versus Salmeterol 50µg After Chronic Obstructive Pulmonary Disease Exacerbation**

**Authors:** *Jill A. Ohar, MD; Glenn D. Crater, MD; Amanda Emmett, MS; Thomas J. Ferro, MD; Andrea N. Morris, BSN; Ibrahim Raphiou, PhD; P.S. Sriram, MD; and Mark T. Dransfield, MD*

Supplemental Material

# Additional file 7: On- and post-treatment serious and fatal adverse events and incidences of pneumonia

On-treatment serious adverse events (SAEs) were reported by 75 (24%) of patients in the fluticasone propionate (FP)/salmeterol (SAL) 250/50µg group and 82 (25%) of patients in the SAL 50µg group. Post-treatment SAEs were reported by 16 (5%) of patients who received FP/SAL and eight (2%) of patients who received SAL. The most frequent on-treatment SAE was chronic obstructive pulmonary disease (COPD; FP/SAL 15%, SAL 16%). The most frequent post-treatment SAE was COPD (FP/SAL 1%, SAL 2%).

Seven patients experienced on-treatment fatal SAEs; four receiving FP/SAL (death due to COPD exacerbation and respiratory failure, anoxic encephalopathy, myoclonic jerking, seizure activity, metabolic acidosis, hypotension and renal failure [all SAEs recorded in one patient on day of death]; worsening of COPD; acute obstructive bronchitis; sudden death) and three receiving SAL (myocardial infarction; cardio-respiratory arrest; septicemia).

A total of 23 patients reported pneumonia AEs post-randomization. As per protocol, all suspected cases of pneumonia were confirmed by chest X-ray. There were 13 (4%) cases of pneumonia in patients receiving FP/SAL and 10 (3%) in patients receiving SAL.
